# Macrophages promote pre-metastatic niche formation of breast cancer through aryl hydrocarbon receptor activity

**DOI:** 10.1038/s41392-024-02042-5

**Published:** 2024-12-18

**Authors:** Xu Jiang, Jiaqi Wang, Liangyu Lin, Liming Du, Yayun Ding, Fanjun Zheng, Hongzhen Xie, Yu Wang, Mingyuan Hu, Benming Liu, Muhan Xu, Jingjie Zhai, Xuefeng Wang, Jiayin Ye, Wei Cao, Chao Feng, Jingyi Feng, Zongliu Hou, Mingyao Meng, Ju Qiu, Qing Li, Yufang Shi, Ying Wang

**Affiliations:** 1 CAS Key Laboratory of Tissue Microenvironment and Tumor, Shanghai Institute of Nutrition and Health, University of Chinese Academy of Sciences, Chinese Academy of Sciences, Shanghai, China; 2 The Third Affiliated Hospital of Soochow University, State Key Laboratory of Radiation Medicine and Protection, Institutes for Translational Medicine, Soochow University, Suzhou, China; 3 The First Affiliated Hospital of Soochow University, Soochow University, Suzhou, China; 4 Key Laboratory of Tumor Immunological Prevention and Treatment of Yunnan Province, Kunming, Yunnan China

**Keywords:** Breast cancer, Immunology

## Abstract

Macrophages that acquire an immunosuppressive phenotype play a crucial role in establishing the pre-metastatic niche (PMN), which is essential for facilitating breast cancer metastasis to distant organs. Our study showed that increased activity of the aryl hydrocarbon receptor (AHR) in lung macrophages plays a crucial role in establishing the immunosuppressive PMN in breast cancer. Specifically, AHR activation led to high expression of PD-L1 on macrophages by directly binding to the promoter of *Pdl1*. This upregulation of PD-L1 promoted the differentiation of regulatory T cells (Tregs) within the PMN, further enhancing immunosuppressive conditions. Mice with *Ahr* conditional deletion in macrophages had reduced lung metastasis of breast cancer. The elevated AHR levels in PMN macrophages were induced by GM-CSF, which was secreted by breast cancer cells. Mechanistically, the activated STAT5 signaling pathway induced by GM-CSF prevented AHR from being ubiquitinated, thereby sustaining its activity in macrophages. In breast cancer patients, the expression of *AHR* and *PD-L1* was correlated with increased Treg cell infiltration, and higher levels of *AHR* were associated with a poor prognosis. These findings reveal that the crosstalk of breast cancer cells, lung macrophages, and Treg cells via the GM-CSF-STAT5-AHR-PD-L1 cascade modulates the lung pre-metastatic niche during breast cancer progression.

## Introduction

Metastatic formation in distant organs is the leading cause of death in breast cancer patients.^1^ The metastatic cascade is a complex, multistep process that involves several critical stages: local invasion of tumor cells into surrounding tissues, intravasation into the bloodstream or lymphatic system, survival during circulation amidst shear forces and immune surveillance, extravasation into distant tissues, and finally, colonization and proliferation to form secondary tumor.^2^ Each of these steps requires cancer cells to adapt to new microenvironments and overcome numerous physical and biological barriers. Emerging evidence suggests that before tumor cells arrive at distant organs, a specialized microenvironment known as the pre-metastatic niche (PMN) is established to support their survival and outgrowth.^3^ These PMNs are characterized by abnormal vascular integrity,^4^ aberrant disposition of extracellular matrix proteins, remodeling of the stroma,^5,6^ and immunosuppression.^7,8^ Factors released from tumor cells, such as cytokines and growth factors, play pivotal roles in mobilizing and recruiting bone marrow-derived cells to regulate the formation of PMN.^7^ Notably, blockade of key molecules in the PMN formation can effectively suppress tumor metastasis in preclinical models.^8^ Therefore, understanding the mechanisms underlying PMN formation is essential for identifying potential therapeutic targets to prevent or reduce metastasis.

Capturing tumor cells and supporting their growth at distant organs also require an immunosuppressive microenvironment that can protect disseminated tumor cells from immune surveillance. Macrophages play a crucial role in forming an immunosuppressive PMN.^7,9^ The activation and accumulation of macrophages in the PMN are driven by multiple factors, including tenascin C,^10^ fibronectin,^11^ complement components,^12^ clot formation,^13^ as well as exosomes^14^ released from tumors. Recently, studies have revealed that these immunosuppressive macrophages in the PMN can be characterized by the expression of triggering receptor expressed on myeloid cells 2 (TREM2) and programmed death-ligand 1 (PD-L1).^14,15^ Under certain conditions, the high expression of PD-L1 in macrophages can promote regulatory T (Treg) cell differentiation,^16,17^ which are critical mediators of immune tolerance and can suppress anti-tumor immune responses. Investigations have shown that Treg cells within the PMN could be generated from CD4^+^ T cells induced by tumor-evoked regulatory B cells.^18^ Additionally, lung carcinoma-derived exosomes have been demonstrated to promote Treg cell differentiation via a CCL1-CCL8 chemokine axis.^19^ Recruitment of Treg cells to the PMN can also be mediated by cyclooxygenase-2 (COX-2)-EP3 signaling and stromal cell-derived factor 1 (SDF-1).^20^ These immune cells collectively remodel and prepare the environment for disseminated tumor cells by suppressing T helper 1 (Th1) cell responses,^12^ inducing T cell exhaustion,^14^ impairing natural killer (NK) cell viability,^21^ and restraining T cell proliferation and cytokine production.^22^

The aryl hydrocarbon receptor (AHR) is a member of the basic helix-loop-helix (bHLH) superfamily of transcription factors.^23^ It functions as a ligand-activated transcription factor and is best known for mediating the toxic effects of environmental contaminants. Upon interaction with exogenous ligands like TCDD (2,3,7,8-tetrachlorodibenzo-p-dioxin)^24^ or endogenous molecules such as kynurenine, a metabolite of the amino acid tryptophan,^25^ AHR translocates from the cytoplasm into the nucleus. In the nucleus, it forms a complex with the AHR nuclear translocator (ARNT), and the AHR-ARNT complex is recruited to gene loci that harbor xenobiotic responsive elements to activate transcription.^26,27^ As a sensor of metabolites and environmental toxins, AHR is recognized as a critical regulator of immune cells, impacting various adaptive immune cells, including CD8^+^ T cells,^28^ CD4^+^ T cells,^25,29^ and B cells,^30^ as well as myeloid cells such as macrophages. In vitro activation of bone marrow-derived macrophages (BMDMs) with kynurenine reduced their expressions of MHC Class I and II, as well as the co-stimulatory molecule, CD86, suggesting an immunosuppressive effect.^31^ In an in vivo glioblastoma model induced by GL261 intracranial injection, the kynurenine produced by glioblastoma cells was found to activate AHR in macrophages, leading to increased expression of CD39. CD39, in cooperation with CD73, can produce adenosine, which promotes CD8⁺ T cell dysfunction and contributes to an immunosuppressive tumor microenvironment.^32^ These studies suggest that AHR is a critical regulator of macrophage functionality. However, how AHR in macrophages reacts specifically to tumor cells and modulates the PMN in breast cancer are not fully understood.

In our study, we observed a significant and temporary increase in AHR activity in lung macrophages prior to breast cancer metastasis. This heightened AHR expression in macrophages led to the generation of Treg cells via PD-L1 upregulation, thereby creating an immune-suppressive environment that promoted tumor cell metastasis. We also found that granulocyte-macrophage colony-stimulating factor (GM-CSF) released from primary breast cancer cells stimulated and stabilized AHR expression through signal transducer and activator of transcription 5 (STAT5) signaling. Consequently, this AHR positively influenced PD-L1 expression by binding to its promoter region, leading to increased transcription. Our findings highlight the crucial role of AHR in lung macrophages in shaping a conducive pre-metastatic environment for breast cancer cells and suggest a potential target to combat metastatic diseases.

## Results

### Changes of AHR activity in lung macrophages prior to breast cancer metastasis

**Fig. 1:**
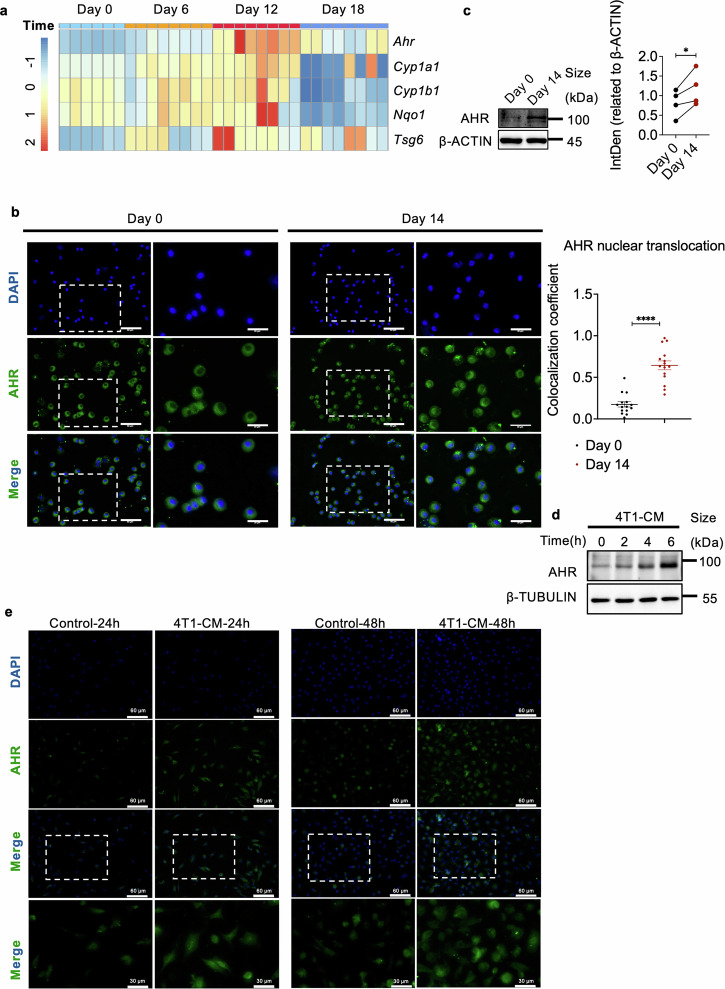
Changes in AHR activity in lung macrophages prior to breast cancer metastasis. **a** Heatmap showing AHR and its downstream gene expression in lung macrophages at indicated timepoints post primary 4T1 tumor establishment. **b** Representative immunofluorescent staining of AHR nuclear translocation in CD45^+^ CD11b^−^ CD11c^+^ SiglecF^+^ macrophages from the lungs of mice bearing 4T1 tumors for 14 days (n = 15 for each group, repeated twice). The scale bars, 60 µm. The scale bars in the enlarged images, 30 µm. The column bars show the coefficient of AHR nuclear translocation in macrophages. **c** Western blotting analysis and quantification of AHR levels in peritoneal macrophages treated with serum from mice bearing 4T1 tumors for 14 days (repeated four times). **d** Western blotting analysis of AHR levels in peritoneal macrophages treated with 4T1-conditioned medium (CM) for indicated times. **e** Representative immunofluorescent staining of AHR nuclear translocation in peritoneal macrophages with or without 4T1-CM treatment. Scale bars, 60 µm. Scale bars in the enlarged images, 30 µm. Data are analyzed by unpaired two-tailed t-test (**b**) or paired two-tailed t-test (**c**) and presented as mean ± SEM (**b**) or symbols & lines (**c**). *p < 0.05, ****p < 0.0001

Macrophages play a key role in promoting tumor growth and metastasis of breast cancer and other malignancies.^33,34^ Their involvement in regulating the formation of the pre-metastatic niche (PMN) is notable, however, the cellular and molecular mechanisms driving their immunosuppressive properties are not well understood. To investigate this, we used the 4T1 mammary carcinoma model, known for its high and spontaneous lung metastasis, to study the properties of lung macrophages before metastatic node formation. After inoculating 4T1 cells into the fat pad, we observed significant lung metastasis around 5 weeks later, with no observable metastasis before day 18 (Supplementary Fig. S1a), which is termed as the stage of PMN. We harvested lung macrophages at various time points post-primary 4T1 tumor inoculation and found a dynamic change in *Ahr* mRNA levels before 4T1 tumor metastasis to the lung (Fig. 1a). Notably, the transcriptional levels of *Ahr* gradually increased at the early stage of breast cancer progression and reached a peak on day 12. We further analyzed the transcriptional levels of major genes downstream of AHR, including cytochrome P450 Family 1 subfamily A member 1 (CYP1A1), cytochrome P450 Family 1 subfamily B member 1 (CYP1B1), NAD(P)H quinone dehydrogenase 1 (NQO1), and TNF alpha-induced protein 6 (TSG6),^35–37^ in lung macrophages of mice inoculated with 4T1 cells (Fig. 1a), showing a consistent trend similar to *Ahr* expression.

Lung macrophages are highly heterogeneous, primarily composed of monocyte-derived macrophages (MDMs), alveolar macrophages (AMs), and interstitial macrophages (IMs). We analyzed their proportions among macrophages in the lung PMN of mice bearing 4T1 tumor and found that AMs were the dominant subpopulation (Supplementary Fig. S1b, c). We also isolated AMs from control mice or mice bearing metastatic 4T1 cells for 14 days and detected nuclear translocation of AHR by immunofluorescent staining (Fig. 1b and Supplementary Fig. S1d) and gene expression profile by RNA-seq (Supplementary Fig. S1e) to characterize the properties of AMs in the PMN. More significant nuclear localization of AHR and AHR associated activity was observed in the AMs of 4T1 tumor-bearing mice, compared to those from tumor-free mice. Interestingly, the expression of AHR and its downstream genes declined at day 18 post-4T1 tumor inoculation (Fig. 1a). These findings suggest that the expression and activation of AHR in lung macrophages may link to breast cancer metastasis.

To investigate the impact of signals from the primary tumor on AHR activity in lung macrophages, we initially treated in vitro cultured macrophages with serum from control mice and mice bearing 4T1 tumors. We observed increased expressions of *Ahr*, *Cyp1b1*, and *Nqo1* in macrophages treated with serum from mice bearing 4T1 tumors (Fig. 1c and Supplementary Fig. S2a, b). To distinguish if serum-derived signals responsible for AHR activation were directly released by 4T1 tumors, we subsequently treated macrophages with 4T1-conditioned medium (CM) and found substantial enhancement in the expression and nuclear translocation of AHR (Fig. 1d, e and Supplementary Fig. S2c–g). Additionally, in an in vitro co-culture of 4T1 cells and macrophages, an upregulation of *Ahr* mRNA level in macrophages was observed when 4T1 cells were seeded at low density (Supplementary Fig. S2h). Similarly, CM from Py8119 cells, another triple-negative breast cancer cell line, also enhanced AHR expression and activity (Supplementary Fig. S2i, j). Taken together, in response to the “signal(s)” from primary tumor cells, the changes of AHR in lung macrophages could be associated with PMN formation during breast cancer progression.

### AHR expressing macrophages promote breast cancer metastasis to the lung via regulating Treg cell differentiation

**Fig. 2:**
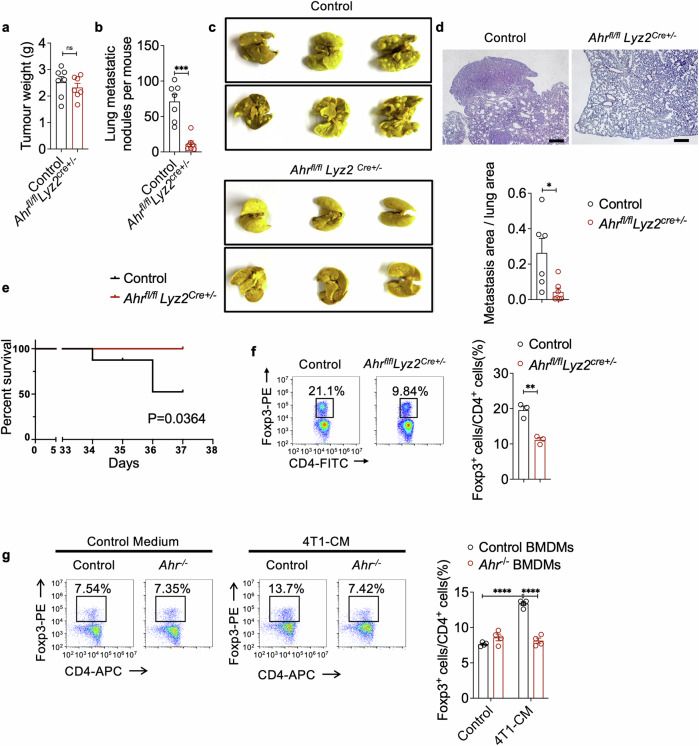
AHR in macrophages influences breast cancer metastasis and Treg cell differentiation. **a**–**c** Wild-type (Control) and *Ahr*^*flfl*^*Lyz2*^*Cre+/−*^ mice were inoculated with 4T1 cells in the mammary gland fat pad for 35 days. Tumor weight (n = 7 for each group) (**a**), the number of macroscopic metastases in the lungs (n = 7 for each group) (**b**), and picric acid-stained lungs (n = 3 for each group) (**c**) were assessed. **d** H&E staining of lung sections from *Ahr*^*flfl*^*Lyz2*^*Cre−/−*^ (Control) and *Ahr*^*flfl*^*Lyz2*^*Cre+/−*^ tumor-bearing mice(n ≥ 6). Scale bars, 400 µm. **e** Survival of 4T1 tumor-bearing wild-type (Control) and *Ahr*^*flfl*^*Lyz2*^*Cre+/−*^ mice (n = 8 for each group). **f** Flow cytometry analysis on Treg cells in the lungs of *Ahr*^*flfl*^*Lyz2*^*Cre−/−*^ (Control) and *Ahr*^*flfl*^*Lyz2*^*Cre+/−*^ mice bearing tumor for 14 days (n = 3 for each group, repeated three times). **g** Flow cytometry analysis on the impact of AHR in macrophages on Treg cell differentiation. BMDMs from wild-type (Control) or *Ahr*^*−/−*^ mice were pretreated with normal medium (Control medium) or 4T1-CM for 2 days. Naïve CD4^+^ T cells were co-cultured with pretreated BMDMs for 3 days under the Treg differentiation conditions (n = 4 for each group, repeated four times). Data are analyzed by unpaired two-tailed t-test (**a**, **b**, **f**, **g**) or Mann Whitney test (**d**), or Log-rank (Mantel-Cox) test (**e**) and presented as mean ± SEM. *p < 0.05, **p < 0.01, ***p < 0.001, ****p < 0.0001, ns, no significance

To explore the contribution of AHR activity in macrophages to breast cancer metastasis to the lung, we employed the Cre-Lox recombination system to specifically delete AHR in macrophages in vivo. *Ahr*^*flfl*^*Lyz2*^*Cre+/−*^ mice and control mice were inoculated with 4T1 cells into the mammary fat pad (Supplementary Fig. S3a). While the primary tumor growth within the mammary fat pad showed no difference (Fig. 2a), we observed that 4T1 cell metastasis to the lung was significantly suppressed in *Ahr*^*flfl*^*Lyz2*^*Cre+/−*^ mice, as indicated by both macroscopic and microscopic analysis of lung tissues (Fig. 2b–d). Consistently, these *Ahr*^*flfl*^*Lyz2*^*Cre+/−*^ mice exhibited a markedly improved survival rate (Fig. 2e). Similar to 4T1 cell metastasis, Py8119 cell metastasis to the lungs was also reduced in *Ahr*^*flfl*^*Lyz2*^*Cre+/−*^ *mice* (Supplementary Fig. S3b).

To determine how AHR activity in macrophages regulates the immunosuppressive PMN formation to facilitate 4T1 tumor colonization and regrowth, we detected immune cell subsets within the lung before metastasis formation. While the levels of CD8^+^ T cells, CD4^+^ T cells, NK cells, B cells, neutrophils, and monocytes were comparable between control mice and *Ahr*^*flfl*^*Lyz2*^*Cre+/−*^ mice (Supplementary Fig. S3c, d and Supplementary Fig. S5c, d), the most prominent change in PMN of *Ahr*^*flfl*^*Lyz2*^*Cre+/−*^ mice was a substantial reduction in the levels of Foxp3^+^ Treg cells (Fig. 2f and Supplementary Fig. S3e). However, the proportions of Treg cells in the primary tumors were not affected by AHR deficiency in macrophages (Supplementary Fig. S3f). Treg cells, a subset of CD4^+^ T cells, suppress immune responses and undermine anti-tumor T cell immunity. An excess of Treg cells has been linked to promoting tumor growth^38,39^ and dissemination,^40,41^ often associated with unfavorable prognoses.^42,43^

To evaluate how AHR-deficient macrophages influence Treg cells, we collected naïve CD4^+^ T cells from the spleens of wild type (WT) mice and co-cultured them with WT BMDMs or *Ahr*^*−/−*^ BMDMs pretreated with either normal medium or 4T1-CM. We found that WT BMDMs treated with 4T1-CM were more effective in inducing Treg cell differentiation compared to those treated with normal medium (Fig. 2g). However, in the absence of AHR, the ability of 4T1-CM pretreated BMDMs to induce Treg differentiation was significantly impaired (Fig. 2g). Notably, when *Ahr*-deficient and control macrophages were pretreated with either LPS or IL-4, their Treg induction efficiencies in the co-culture system were comparable (Supplementary Fig. S3g, h). This specific regulation of *Ahr*-activated macrophages in Treg cell induction occurs in breast cancer conditions. In addition to *Ahr*^*flfl*^*Lyz2*^*Cre+/−*^ mice, we also performed 4T1 inoculations in AHR-deficient (*Ahr*^*−/−*^) mice and evaluated their lung metastasis. Similar to the breast cancer progression in *Ahr*^*flfl*^*Lyz2*^*Cre+/−*^ mice, *Ahr*^*−/−*^ mice showed a reduction in metastatic nodes, a decrease in Treg cell levels within the lungs, but no change in primary tumor growth (Supplementary Fig. S3i–l). These results suggest that AHR activation in macrophages could modulate the immunosuppression of the PMN and influence the spread of breast cancer to the lungs.

### PD-L1 mediates the promotion of Tregs by AHR activated macrophages

**Fig. 3:**
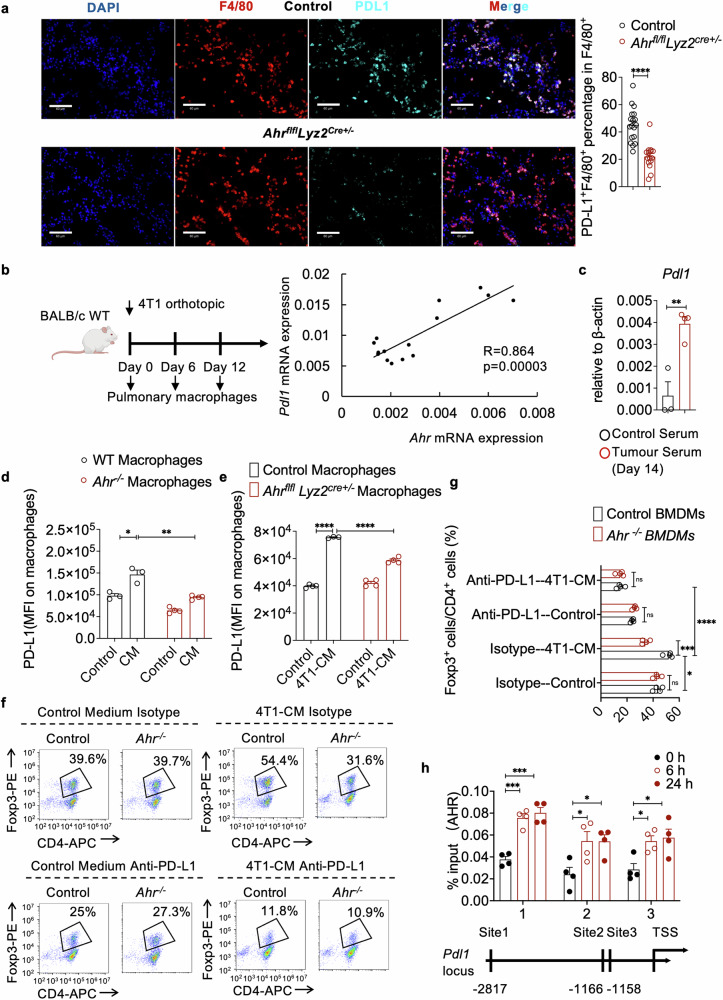
Promotion of Treg cells by AHR activated macrophages through PD-L1. **a** Representative immunofluorescent images showing F4/80 and PD-L1 staining in the lungs of *Ahr*^*flfl*^*Lyz2*^*Cre−/−*^ (Control) and *Ahr*^*flfl*^*Lyz2*^*Cre+/−*^ mice inoculated with 4T1 cells for 14 days (n ≥ 15 for each group). Scale bar, 60 μm. **b** Schematic of lung macrophage isolation from mice bearing 4T1 tumors at indicated timepoints (created with BioRender.com). Correlation analysis between *Pdl1* and *Ahr* mRNA expressions in F4/80^+^ cells from the PMN (n = 15 for each group). **c** *Pdl1* mRNA expression in peritoneal macrophages, with or without addition of serum from 4T1 tumor bearing mice (n ≥ 3 for each group, repeated twice). **d** PD-L1 expression on peritoneal macrophages of wild-type (WT) and *Ahr*^*−/−*^ mice, with or without 4T1-CM treatment for 12 h (n ≥ 3 for each group). **e** PD-L1 expression on peritoneal macrophages from *Ahr*^*flfl*^*Lyz2*^*Cre−/−*^ (Control) and *Ahr*^*flfl*^*Lyz2*^*Cre+/−*^ mice, with or without 4T1-CM (n ≥ 3 for each group, repeated twice). **f** Flow cytometry analysis of Treg cell differentiation when co-cultured with normal medium or 4T1-CM pretreated macrophages from WT mice and *Ahr*^−/−^ mice in the presence of isotype control or anti-PD-L1. BMDMs from WT mice (Control) or *Ahr*^*−/−*^ mice were pretreated with normal medium (Control) or 4T1-CM for 2 days. Naïve CD4^+^ T cells were co-cultured with pretreated BMDMs for 3 days under the Treg cell differentiation conditions, with the addition of PD-L1 neutralizing antibody or Isotype control. **g** Bar graph showing quantification of Treg cell differentiation (n = 4 for each group). **h** ChIP analysis of AHR recruitment to the *Pdl1* promoter in peritoneal macrophages stimulated with 4T1-CM (n = 4 for each group). Data are analyzed by unpaired two-tailed t-test (**a**, **c**, **d**, **e**, **g**, **h**) or Pearson correlation analysis (**b**) and presented as mean ± SEM. *p < 0.05, **p < 0.01, ***p < 0.001, ****p < 0.0001, ns, no significance

Macrophages exert immunosuppressive functions through expression of anti-inflammatory molecules, such as PD-L1.^44^ In 4T1-bearing *Ahr*^*flfl*^*Lyz2*^*Cre+/−*^ mice, we observed a significant decrease of PD-L1 expression on macrophages from the PMN, but not from the primary tumor sites (Fig. 3a and Supplementary Fig. S4a, b). A positive correlation between *Pdl1* and *Ahr* expression was also evident in F4/80-positive cells within the PMN during 4T1 tumor progression (Fig. 3b). Notably, in the PMN, PD-L1 was mainly expressed on AMs (Supplementary Fig. S5a, b). Meanwhile, upon treatment with serum of 4T1-bearing mice or 4T1-CM, PD-L1 expression in macrophages was upregulated (Fig. 3c–e and Supplementary Fig. S4c, d). This elevated PD-L1 expression was reduced in AHR-deficient macrophages and suppressed by CH223191, an AHR inhibitor (Supplementary Fig. S4c–e). To generalize these findings to other breast cancer types, we also analyzed *Pdl1* expression in macrophages from the PMN of mice inoculated with Py8119 and in macrophages treated with Py8119-CM. Similar to what was observed in 4T1 cell treatment, PD-L1 expression in Py8119-treated macrophages was also increased (Supplementary Fig. S4f, g). Since *Ahr* deficiency in myeloid cells may influence monocytes and neutrophils, we also analyzed PD-L1 expression on these cells in the PMN of *Ahr*^*flfl*^*Lyz2*^*Cre+/−*^ mice and control mice bearing breast cancer and found their PD-L1 expression was not affected (Supplementary Fig. S5c, d). Thus, the positive regulatory role of AHR on PD-L1 level during breast cancer progression specifically occurs in lung macrophages.

PD-L1, kno*w*n for its role in promoting Treg cell differentiation,^16,17^ was investigated in relation to macrophages. When PD-L1 neutralizing antibodies were introduced to the co-culture system of naïve CD4^+^ T cells and BMDMs from control or *Ahr*^*−/−*^ mice, the promotion of Treg cells by 4T1-CM treated macrophages was effectively inhibited, indicating that PD-L1 expression in macrophages plays a key role in promoting Treg cell differentiation (Fig. 3f, g).

We next explored how AHR controls the expression of PD-L1 in macrophages. Previous studies have demonstrated that AHR is a transcription factor and forms a heterodimer with ARNT to induce transcription of various target genes.^26,27^ To ascertain whether *Pdl1* is among the genes targeted by AHR, we conducted a Chromatin Immunoprecipitation-quantitative Polymerase Chain Reaction (ChIP-qPCR) experiment in macrophages and successfully confirmed the binding of AHR to the promoter region of *Pdl1* following 4T1-CM treatment (Fig. 3h), suggesting that AHR activation in macrophages promotes Treg cell levels in a PD-L1-dependent manner.

### GM-CSF from breast cancer cells regulates AHR expression in macrophages

**Fig. 4:**
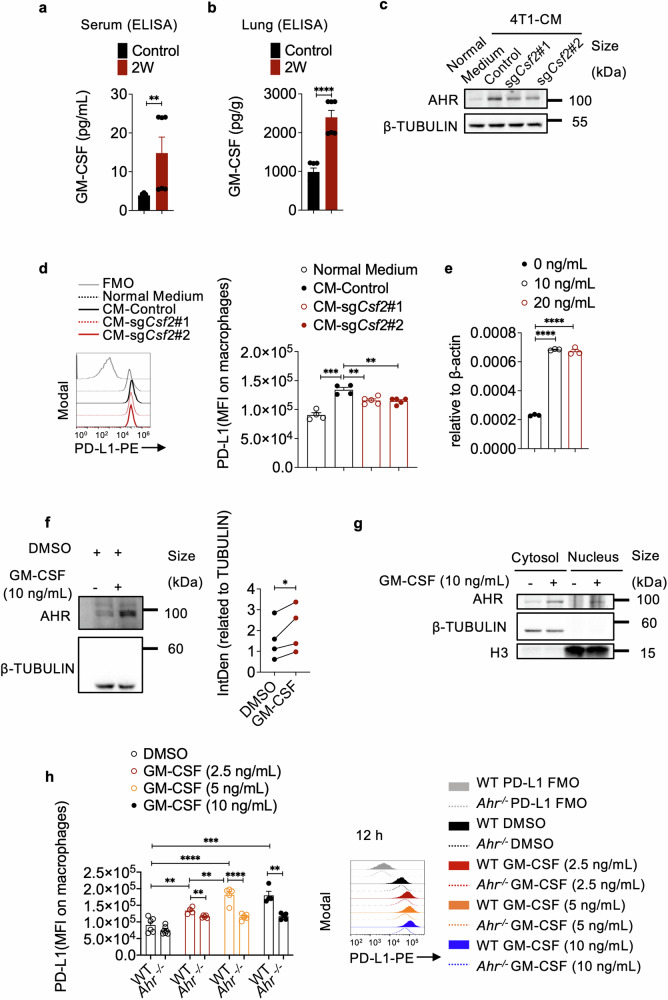
GM-CSF released from breast cancer cells promotes AHR expression in macrophages. Quantification of GM-CSF levels in both serum (**a**) and lung tissue (**b**) of mice inoculated with 4T1 cells in the mammary gland fat pad for 2 weeks using ELISA (n = 6 for each group). **c** AHR expression in peritoneal macrophages cultured in normal medium, or CM from control 4T1 cells, or sg*Csf2* transfected 4T1 cells for 12 h (repeated twice). **d** Histogram showing PD-L1 expression on peritoneal macrophages cultured in normal medium, CM from control 4T1 cells, or CM from sg*Csf2*-transfected 4T1 cells for 24 h (n ≥ 4 for each group, repeated three times). The bar graph shows MFI of PD-L1 on each group of macrophages. **e** mRNA expression of *Ahr* in peritoneal macrophages treated with GM-CSF at indicated concentrations for 24 h (n = 3 for each group, repeated twice). **f** Western blotting analysis and quantification of AHR expression in peritoneal macrophages treated with GM-CSF (10 ng/mL) for 24 h. **g** Western blotting analysis of AHR expression in the cytoplasm and nucleus of macrophages treated with or without GM-CSF. **h** Histogram showing PD-L1 expression on WT or *Ahr*^*−/−*^ peritoneal macrophages treated with or without GM-CSF (2.5, 5, or 10 ng/mL) for 12 h. The bar graph shows MFI of PD-L1 on each group of macrophages (n ≥ 4 for each group). Data are analyzed by unpaired two-tailed t-test (**b**, **d**, **e**, **h**) or Mann Whitney test (**a**) or paired two-tailed t-test (**f**) and presented as mean ± SEM (**a**, **b**, **d**, **e**, **h**) or symbols & lines (**f**). *p < 0.05, **p < 0.01, ***p < 0.001, ****p < 0.0001

As soluble factors derived from 4T1 cells were found to significantly stimulate AHR activity in macrophages, we sought to identify the key factor responsible for these changes and their impact on PMN formation. Using centrifugal filters to fractionate 4T1-CM based on molecular weights, we determined that the soluble factor influencing AHR regulation has a molecular weight larger than 3 kDa (Supplementary Fig. S6a), ruling out the possibility of small molecules such as metabolites. Further investigation led to the discovery that granulocyte-macrophage colony-stimulating factor (GM-CSF) with a molecular weight of ~14 kDa was abundant in 4T1-CM.^45^ Its level in the serum and lungs of 4T1 tumor-bearing mice on day 14 was notably increased (Fig. 4a, b and Supplementary Fig. S6b).

To directly examine the influence of GM-CSF from 4T1 cells on macrophages, we used lentiCRISPR v2 plasmids expressing cas9 protein and GM-CSF-targeting sgRNA to create GM-CSF deficient 4T1 cells (Supplementary Fig. S6c). The CM collected from these GM-CSF deficient 4T1 cells lost its ability to increase AHR and PD-L1 expressions in macrophages (Supplementary Fig. S6d, e). To minimize potential effects related to lentivirus genome insertion, we also utilized recombinant Cas9 protein and synthesized sgRNA to establish GM-CSF-deficient 4T1 cells (Supplementary Fig. S6f), further confirming that GM-CSF mediates the impact of 4T1 cells on the expressions of AHR and PD-L1 in macrophages (Fig. 4c, d). Additionally, treating macrophages with recombinant GM-CSF led to a significant increase in AHR expression and activity in macrophages (Fig. 4e–g). Importantly, the absence of AHR in macrophages eliminated the ability of GM-CSF to enhance PD-L1 expression (Fig. 4h and Supplementary Fig. S6g, h). These findings demonstrate a connection between the primary 4T1 tumor and macrophages in the PMN through a GM-CSF-AHR-PD-L1 axis.

### STAT5 activation induced by GM-CSF enhances AHR stability via inhibiting its ubiquitination

**Fig. 5:**
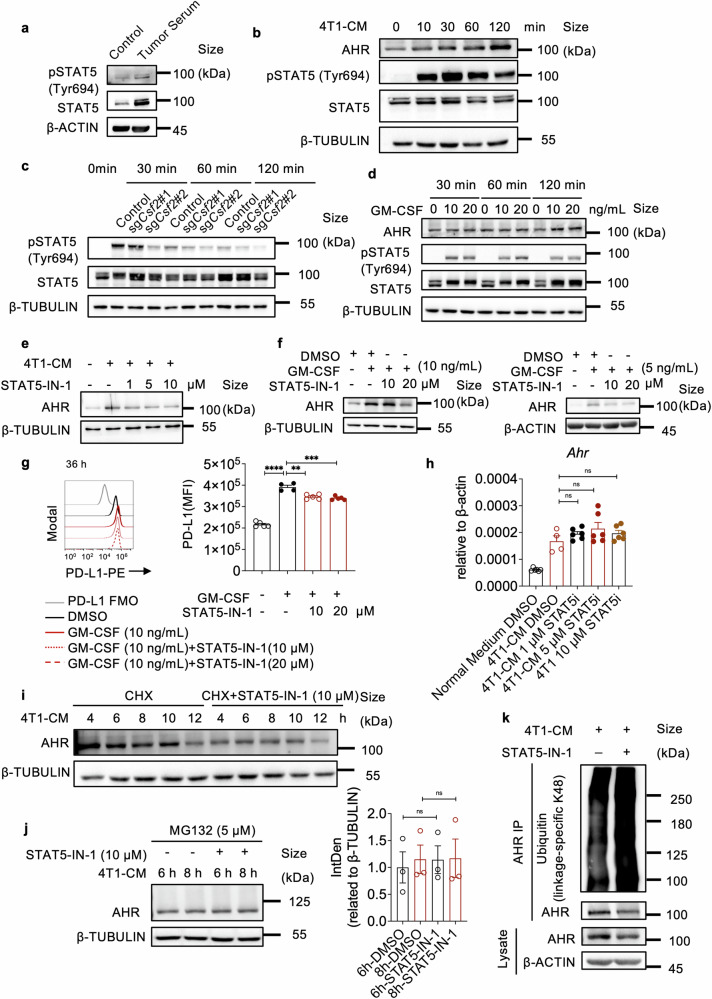
GM-CSF-initiated STAT5 signaling enhances AHR stability by inhibiting AHR ubiquitination. **a** Western blotting analysis of pSTAT5 and STAT5 expressions in peritoneal macrophages treated with serum from mice inoculated with or without 4T1 cells for 14 days (repeated twice). **b** Western blotting analysis of AHR, pSTAT5, and STAT5 levels in peritoneal macrophages treated with 4T1-CM at indicated time points (repeated three times). **c** Western blotting analysis of pSTAT5 and STAT5 expressions in peritoneal macrophages treated with CM from control 4T1 cells or *Csf2* knockout 4T1 cells at indicated time points. **d** Western blotting analysis of AHR, pSTAT5, and STAT5 expressions in peritoneal macrophages treated with GM-CSF (0, 10, 20 ng/mL) for 30 min, 60 min, and 120 min. **e** Western blotting analysis of AHR expression in STAT5-IN-1-treated peritoneal macrophages, with or without 4T1-CM treatment for 24 h (repeated twice). **f** Western blotting analysis of AHR expression in peritoneal macrophages treated with GM-CSF (10 ng/mL or 5 ng/mL), with or without the addition of STAT5-IN-1 (10 or 20 μM) (repeated twice). **g** Histogram of PD-L1 expression on peritoneal macrophages treated with DMSO or GM-CSF (10 ng/mL) for 36 h, with or without the addition of STAT5-IN-1 (10 or 20 μM) (n ≥ 4 for each group, repeated twice). The bar graph shows MFI of PD-L1 on each group of macrophages. **h** mRNA levels of *Ahr* in peritoneal macrophages treated with or without 4T1-CM, in the presence or absence of STAT5-IN-1 (1, 5, or 10 μM) for 24 h (n ≥ 4 for each group, repeated three times). **i** The protein levels of AHR in peritoneal macrophages treated with 4T1-CM and CHX (5 µg/ml), with or without the addition of STAT5-IN-1 (10 μM) (repeated three times). **j** The protein levels and quantification of AHR in peritoneal macrophages treated with 4T1-CM and MG132 (5 μM) for 6 or 8 h, with or without the addition of STAT5-IN-1 (10 μM). **k** Western blotting analysis of ubiquitin in macrophages treated with 4T1-CM and STAT5-IN-1. Peritoneal macrophages cultured in 4T1-CM were treated with DMSO or STAT5-IN-1 (10 μM) for 12 h. The cell lysates were immunoprecipitated with an anti-AHR antibody and immunoblotted with an anti-ubiquitin antibody (repeated three times). Data are analyzed by unpaired two-tailed t-test (**g**, **h**, **j**) and presented as mean ± SEM. **p < 0.01, ***p < 0.001, ****p < 0.0001, ns no significance

After GM-CSF binds to its receptor, the activation of STAT5 is a key signaling pathway that regulates various genes to impact cell properties.^46,47^ Significant activation of STAT5 was observed in macrophages treated with the serum of 4T1 tumor-bearing mice (Fig. 5a). Additionally, 4T1-CM could induce STAT5 activation in macrophages (Fig. 5b). STAT5 activation was also observed in macrophages treated with Py8119-CM (Supplementary Fig. S7g). Using CM derived from GM-CSF-deficient 4T1 cells to treat macrophages resulted in impaired STAT5 phosphorylation (Fig. 5c and Supplementary Fig. S7a), indicating the reliance of breast cancer cells on GM-CSF to promote STAT5 activation in macrophages. Furthermore, GM-CSF alone can significantly enhance AHR expression, accompanied by the upregulation of STAT5 phosphorylation (Fig. 5d). When specific inhibitors of STAT5 phosphorylation, such as STAT5-IN-1 and IQDMA, were added to 4T1-CM treated macrophages, a dramatic reduction in AHR expression and nuclear translocation can be observed (Fig. 5e and Supplementary Fig. S7b–d). Similarly, the addition of STAT5-IN-1 to in vitro cultured macrophages effectively attenuated GM-CSF-induced AHR and PD-L1 expressions (Fig. 5f, g). We also used STAT5-IN-1 to treat 4T1-bearing mice and found that AHR expression in lung macrophages within the PMN was decreased (Supplementary Fig. S7e). These findings suggest that breast cancer cells enhance AHR expression in macrophages through GM-CSF and its induction of STAT5 signaling.

STAT5 is a well-known transcription factor, and phosphorylated STAT5 can translocate to the nucleus to activate the transcription of its downstream genes.^48,49^ In our study, we observed that the STAT5 inhibitor could reduce AHR protein levels in macrophages treated with 4T1-CM, without significantly affecting *Ahr* mRNA levels (Fig. 5h). These findings strongly indicate that STAT5 signaling does not influence the transcription of *Ahr*. To investigate whether phosphorylated STAT5 affects AHR through post-transcriptional mechanisms, we introduced cycloheximide (CHX), a protein-synthesis inhibitor, to macrophages treated with 4T1-CM in the presence and absence of the STAT5 inhibitor. The protein level of endogenous AHR was still notably decreased by STAT5-IN-1 (Fig. 5i), indicating that STAT5 phosphorylation controls AHR protein levels by impeding AHR protein degradation. Additionally, when Z-leu-leu-leu-al (MG132) was used to block proteasome-mediated protein degradation, the impact of STAT5-IN-1 on AHR protein levels was neutralized (Fig. 5j). Moreover, treatment with STAT5-IN-1 led to a reduction in AHR protein levels in 4T1-CM-treated macrophages, and this effect was partially restored by the addition of MG132 (Supplementary Fig. S7f). Importantly, STAT5-IN-1 intensified the ubiquitination of AHR (Fig. 5k). In summary, triggering STAT5 signaling bolsters AHR stability and expression by preventing its ubiquitination.

### The *AHR*-*PD-L1*-Tregs and the prognosis of breast cancer in patients

**Fig. 6:**
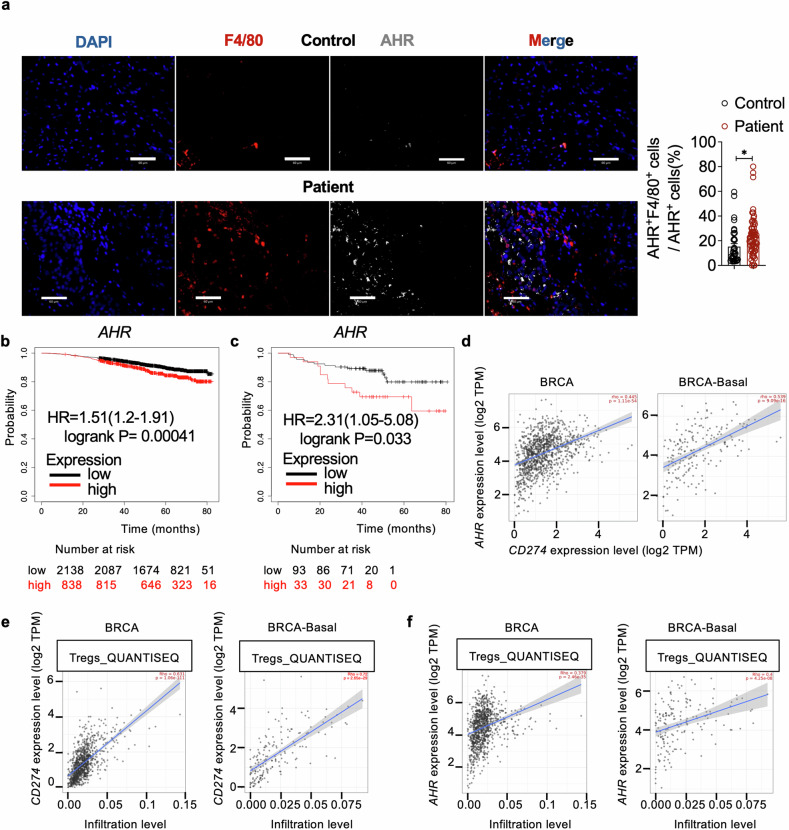
The prognosis value of the *AHR*-*PD-L1*-Tregs axis in breast cancer patients. **a** Representative immunofluorescent images showing F4/80 and AHR staining in the lungs of patients and control. Scale bars, 60 µm. The bar graph shows the percentage of AHR⁺ F4/80⁺ cells among AHR⁺ cells (n ≥ 35 for each group). **b** Overall survival of breast cancer patients with high and low *AHR* expression. **c** Overall survival of TNBC patients with high and low *AHR* expression. *AHR* expression and breast cancer patient survival analysis using the Kaplan-Meier Plotter online tool (http://kmplot.com/analysis/). **d** The correlation between *AHR* and *PD-L1* (*CD274*) expression in breast cancer and its subtype, basal-like breast cancer. **e** The correlation between Treg cell infiltration and *PD-L1* expression in breast cancer. **f** The correlation between Treg cell infiltration and *AHR* expression in breast cancer. The correlation analysis among *AHR*, *PD-L1*, and Treg cells was analyzed using the TIMER2.0 online tool (http://timer.cistrome.org/). Data are analyzed by unpaired two-tailed t-test (**a**) and presented as mean ± SEM. *p < 0.05

To investigate the clinical relevance of our findings, we assessed AHR⁺ F4/80⁺ macrophages in patients suffering breast cancer lung metastasis. Our results indicate that lung specimens from metastatic patients show a significant increase in the percentage of AHR⁺F4/80⁺ cells among AHR^+^ cells (Fig. 6a). Additionally, our analysis using the Kaplan-Meier Plotter online tool showed a correlation between *AHR* expression and the overall survival (OS) of breast cancer patients. Patients with low *AHR* expression exhibited better OS compared to those with higher *AHR* expression, suggesting a potential role of *AHR* in diagnosing breast cancer progression (Fig. 6b). This correlation was also evident in triple-negative breast cancer (TNBC), the most aggressive subtype of breast cancer characterized by the absence of Estrogen Receptor (ER), Progesterone Receptor (PR), and HER2 (Fig. 6c). Furthermore, we found a positive relationship between *AHR* and *PD-L1* (*CD274*) expressions in breast cancer patients, particularly in those with basal-like breast cancer (Fig. 6d), which is a subtype of TNBC.^50^ These findings support the connection between AHR and PD-L1 in the mouse lung PMN of breast cancer. Moreover, in-depth analysis of breast cancer, especially the basal-like breast cancer for the correlation between the levels of *PD-L1* (Fig. 6e) or *AHR* (Fig. 6f) and the infiltration of Treg cells revealed that high levels of *PD-L1* and *AHR* were associated with increased infiltration of Treg cells in patients. This positive link among *AHR*, *PD-L1*, and Treg cell infiltration correlates with poor prognosis for breast cancer.

## Discussion

Numerous cellular components and factors have been identified as contributors to the formation of the pre-metastatic niche, a crucial environment that aids in the metastasis of breast cancer, particularly to the lungs. Understanding the PMN is essential because metastasis is the leading cause of cancer-related deaths, and the lung is a common site for breast cancer metastasis. Despite advances in cancer therapy, the mechanisms underlying PMN formation and its immunosuppressive nature remain largely elusive. In a mouse model of breast cancer, our research has uncovered a significant role for the AHR in lung macrophages, which is induced by GM-CSF secreted by 4T1 breast cancer cells. This pathway plays a crucial role in creating an immunosuppressive microenvironment that allows breast cancer cells to localize, survive, and grow in the lungs. Our findings highlight a novel mechanism by which tumor-derived factors manipulate the immune landscape of distant organs, facilitating metastasis.

Activation of AHR in macrophages was shown to facilitate the differentiation of Treg cells through its upregulation of PD-L1 expression, with its influence on PD-L1 dependent on the direct binding of AHR to the promoter of *Pdl1*. This suggests that AHR not only modulates macrophage function but also has downstream effects on T cell regulation, contributing to an immunosuppressive milieu. Additionally, GM-CSF released by 4T1 cells regulates AHR expression in macrophages via STAT5 signaling. Our data suggest that STAT5 stabilizes and enhances AHR levels by inhibiting its ubiquitination, thereby prolonging the presence of AHR in the macrophages. This post-translational regulation of AHR by STAT5 represents a previously unrecognized mechanism of immune modulation in the PMN.

The importance of AHR, PD-L1, and Tregs in this context also provides a valuable prognostic indicator for breast cancer, given that these factors are correlated with patient outcomes. We propose that AHR in lung macrophages serves as a crucial switch in determining the immunosuppressive characteristics of the PMN, particularly during the early stages of metastasis. Several key observations support this conclusion. First, we observed dynamic changes in AHR levels in lung macrophages before the appearance of visible metastatic nodules. During breast cancer progression, AHR expression in lung macrophages peaked at ~12 days after 4T1 cell inoculation, followed by a subsequent decrease. Second, we found that *Ahr*^*flfl*^*Lyz2*^*Cre+/−*^ mice exhibited resistance to breast cancer metastasis to the lungs, while the growth of the primary tumor remained unaffected. This result strongly supports the role of AHR in lung macrophages as a pivotal factor in the metastatic process. Third, in vitro co-culture experiments demonstrated that when 4T1 cells were present at low ratios relative to macrophages, AHR expression was significantly enhanced in macrophages. However, as the number of 4T1 cells increased, the level of AHR in macrophages decreased. Consistently, macrophages isolated from the lungs at the late stage of PMN presented lower levels of *Ahr* than those from early and mid-stages of PMN. These findings suggest a temporal regulation of AHR expression in macrophages, influenced by tumor cell interactions, which may be critical in the initiation of metastasis. We agree that its effects are likely pleiotropic and that our data did not unequivocally show the effects of high number of breast cancer cells on *Ahr* expression in macrophages which are not linked to GM-CSF or related to other potential regulation. Further investigation is required to elucidate the mechanisms by which 4T1 cells dynamically influence AHR expression in macrophages, particularly at different stages of tumor progression.

The present findings also indicate that GM-CSF produced by 4T1 cells plays a critical role in upregulating AHR expression in macrophages of distant organs. Its promotion of AHR expression occurs through a STAT5-mediated post-translational mechanism. Previous studies have demonstrated that tryptophan derivatives, such as kynurenine and kynurenic acid, can activate AHR in various cancer models. For instance, in a glioma mouse model, kynurenine produced by tumor cells was found to directly activate AHR in tumor-associated macrophages.^32^ This process was facilitated by the expression of tryptophan 2,3-dioxygenase (TDO-2) and indoleamine 2,3-dioxygenase-1 (IDO-1) enzymes, which are involved in kynurenine production. However, it is worth noting that the levels of kynurenine-catabolizing enzymes, including *Ido1* and *Tdo2*, in 4T1 cells were not higher than those observed in macrophages (Supplementary Fig. S6i), suggesting that kynurenine is not the major ligand responsible for AHR activation in this system. Alternatively, it is possible that the increased AHR levels in macrophages within the PMN make them more susceptible to activation, and the ligand responsible for AHR activation may not necessarily originate from 4T1 cells, as endogenous AHR ligands have diverse sources.^51,52^ Nonetheless, our research highlights the critical role of AHR in shaping the immunosuppressive microenvironment that facilitates breast cancer metastasis to the lungs.

STAT5 plays a crucial role in regulating the responsiveness of immunogenic stimulations in T cells and macrophages.^53–55^ Our study has uncovered a previously unrecognized post-translational modulation mechanism through which STAT5 influences AHR and its related immunoregulation. We observed that STAT5 signaling effectively inhibits the ubiquitination of AHR, thereby enhancing AHR stability and expression. Since STAT5 is known to mediate various cytokine activation signals, including IL-2,^56^ and recent research has shown that IL-2 can stabilize AHR protein in CD8^+^ T cells,^57^ it is possible that post-translational regulation by STAT5 is conserved in both adaptive and innate immune cells. Therefore, a comprehensive investigation into the molecular mechanisms underlying STAT5 signaling in the regulation of the proteasome will enhance our understanding of the changes in AHR in macrophages within the PMN of breast cancer.

Our investigation on the role of AHR in the PMN of breast cancer has yielded new insights into AHR-mediated regulation of tumor metastasis. It’s noteworthy that deletion of AHR in macrophages strongly correlates with the prevention of breast cancer metastasis to the lung, indicating a promising target for controlling cancer metastasis.^8^ Recently, clinical trial results of the AHR inhibitor, BAY 2416964, in the treatment of various solid tumors have not met anticipated success (ClinicalTrials.gov ID: NCT04069026). Therefore, identifying the appropriate stages or windows for the use of AHR inhibitors, such as post-surgery and high-risk pre-metastasis stages where GM-CSF levels might serve as a valuable marker, will be crucial and practical. Tailoring AHR-targeted therapies to specific stages of cancer progression could enhance their efficacy and reduce unintended effects on normal immune function. Collectively, this study suggests that the increased AHR levels in lung macrophages, induced by GM-CSF from primary breast cancer, are crucial for establishing an immunosuppressive environment for breast cancer cell colonization and regrowth. We propose that AHR in the PMN may be a potential therapeutic target to limit breast cancer lung metastasis. Future research focusing on AHR modulation in the PMN could pave the way for novel interventions to prevent or treat metastatic breast cancer.

## Materials and methods

### Mice and human subjects

All human tissues were collected with informed consent, following approval by the Ethics Committee of the First Affiliated Hospital of Soochow University (no. SUFAH-2019-141). There were 5 breast cancer patient samples and 3 control samples. Clinical details were recorded in Supplementary Table 4.

Mice were maintained in a specific pathogen-free (SPF) facility of the Shanghai Institute of Nutrition and Health of the Chinese Academy of Sciences and used in accordance with the ethical guidelines of the Institutional Animal Care and Use Committee.

Wild-type (WT) mice, with a BALB/c background, aged 8–10 weeks, were obtained from the Shanghai Laboratory Animal Center of the Chinese Academy of Sciences. C57BL/6 strain *Lyz2*^*Cre*^ mice and C57BL/6 strain *Ahr*^*fl/fl*^ mice were purchased from the Jackson Laboratory, USA. C57BL/6 strain *Ahr*^*–/–*^ mice were generated previously.^58^ C57BL/6 strain *Ahr*^*fl/fl*^*Lyz2*^*Cre*^ mice were generated through the crossing of C57BL/6 strain *Ahr*^*fl/fl*^ mice with C57BL/6 strain *Lyz2*^*Cre*^ mice. BALB/c strain *Ahr*^*−/−*^ mice were produced by backcrossing C57BL/6 strain *Ahr*^*−/−*^ mice with BALB/c wild-type mice for 8 generations. BALB/c strain *Ahr*^*fl/fl*^*Lyz2*^*Cre*^ mice were generated by further crossing C57BL/6 strain *Ahr*^*fl/fl*^*Lyz2*^*Cre*^ mice with BALB/c wild-type mice for 8 generations.

### Cell isolation and culture

The 4T1 cell line (cat. no. CRL-2539) was purchased from ATCC. Peritoneal macrophages were harvested from BALB/c mice *i.p*. injected with 2 mL of 5% (wt/vol) thioglycollate broth media (BD, B11716) for 5 days. Bone marrow-derived macrophages were generated from femur and tibia bone marrows of BALB/c mice. Bone marrow cells were cultured in medium containing 20 ng/mL M-CSF (GenScript, Z02930-50) for 6 days. Mouse CD4^+^ T cells were isolated from splenocytes using immunomagnetic separation beads (Miltenyi Biotec, 130-104-454). F4/80^+^ cells were isolated from lung tissue suspension using immunomagnetic separation beads (Invitrogen, 8802-6863-74). All cells were grown in RPMI 1640 (C11875500CP) complete medium, containing 10% FBS (EallBio, 03.A16001DC), 2 mM glutamine (Gibco, 35050061), and 100 U/mL penicillin-streptomycin (Gibco, 15140122). The Py8119 cell line was kindly provided by Dr. Guohong Hu of Shanghai Institute of Nutrition and Health of Chinese Academy of Sciences. Py8119 cells were cultured in DMEM medium (Gibco,11995073), containing 10% FBS (EallBio, 03.A16001DC), 100 U/mL penicillin-streptomycin (Gibco, 15140122), and 2 mM glutamine (Gibco, 35050061).

### Mouse 4T1 tumor models

For the pulmonary metastatic model, 5 × 10^5^ to 8 × 10^5^ 4T1 cells suspended in 100 µL PBS were injected into the right mammary fat pad of BALB/c mice. Experiments were typically ended after 5 weeks unless otherwise stated. The serum and lungs were collected and processed following the ex vivo methods described. To inhibit STAT5 in vivo, STAT5 inhibitor (MCE, HY-452 101853-5 mg) or DMSO (Sigma, D2650) was diluted in 100 μl PBS and injected *i.p*. administered at a dose of 200 μg/mouse on day −3, −1, 1, 3, 5, 7, 9, 11, and 13 before and after mice inoculating with 4T1 cells into the fat pad. STAT5 inhibitors were prepared in a DMSO stock solution and further mixed with PBS prior to in vivo administration.

For the Py8119 tumor model, 5 × 10^4^ to 1 × 10^5^ Py8119 cells were mixed with Matrigel (Corning biocoat, 356237) and injected into the mammary fat pads of 8-week-old male mice. Surgical resection of the tumor was performed 4 weeks later, experiments were typically ended after another 3 weeks.

### RNA isolation and RT-qPCR

Total RNA was extracted using the Trizol reagent (Invitrogen, 15596018) and RNAprep pure cell/bacteria kit (TIANGEN, DP430), following the manufacturers’ instructions. cDNA was synthesized using the RT Master Mix kit (Takara, RR036A). Relative mRNA expression was determined by real-time qPCR. The FastStart Universal SYBR Green Master kit (Roche, 4913914001), cDNA, and primers were performed. Primer sequences are as follows: mouse *Actb* : forward 5′- TTCCAGCCTTCCTTCTTGGG-3′ reverse 5′- TGTTGGCATAGAGGTCTTTACGG-3′; mouse *Ahr* : forward 5′- CCGAAGCACACGCAAATCAA-3′ reverse 5′- CCCTTCCAGGGAAGTCCAAC-3′; mouse *Nqo1*: forward 5′-AGGATGGGAGGTACTCGAATC′ reverse 5′- AGGCGTCCTTCCTTATATGCTA-3′; mouse *Cyp1a1*: forward 5′- ACAGACAGCCTCATTGAGCA-3′ reverse 5′- GGCTCCACGAGATAGCAGTT-3′; mouse *Cyp1b1*: forward 5′- CACCAGCCTTAGTGCAGACAG-3′ reverse 5′- GAGGACCACGGTTTCCGTTG-3′; mouse *Tsg6*: forward 5′- GGGATTCAAGAACGGGATCTTT-3′ reverse 5′- TCAAATTCACATACGGCCTTGG-3′; mouse *Pdl1*: forward 5′- GCTCCAAAGGACTTGTACGTG-3′ reverse 5′- TGATCTGAAGGGCAGCATTTC-3′.

### Western blot and immunoprecipitation

Cells were rinsed with PBS and then lysed in RIPA buffer (Beyotime, P0013B) containing PI (Roche, 04693116001) and PMSF on ice for 30 min. The protein concentration of each sample was determined using a BCA Protein Assay kit (Thermo Scientific, 23225). Protein samples (20–30 μg) were loaded onto SDS–PAGE gels and transferred onto a nitrocellulose membrane. After blocking with 5% fat-free milk or 5% BSA in Tris-buffered saline with 0.1% (w/v) Tween 20 at room temperature for 1 h, nitrocellulose membranes were incubated with the indicated primary and secondary antibodies, following the manufacturer’s protocols.

For the Immunoprecipitation assay, samples were rotated with the antibody against AHR (Enzo, BML-SA210-0100) at 4 °C overnight. Protein G agarose beads (Roche, 11243233001) were then added, and incubation continued for 4 h. The beads (30 µl) were spun down and washed three times with cold PBS. Samples were reconstituted in 1× loading buffer and heated to 100 °C for 10 min.

For the ubiquitination assay, prior to RIPA collection, cells were incubated with 5 μM MG132 (Sigma, C2211-5MG) for 4 h. Samples were then incubated with an anti-Ubiquitin antibody (Abcam, ab140601) at 4 °C overnight, followed by washes and processed for western blot analysis.

For nuclear-cytoplasmic separation assay, cells were collected and washed 3 times with pre-cooled 1 × PBS to estimate the volume of cell precipitation. Five times the volume of pressure-reducing buffer was added and placed on ice for 10 min. The cells were subjected to centrifugation at 3000 × *g* for 5 min at 4 °C, the supernatant was collected and again subjected to centrifugation at 6000 × *g* for 10 min at 4 °C, The resulting supernatant was considered as the cytoplasmic portion. The precipitate portion of the first centrifugation was washed twice with hypotonic buffer. The precipitate was considered as the nucleus portion.

### Hematoxylin and eosin staining

Lungs from 4T1 tumor-bearing mice were collected and fixed in 4% paraformaldehyde overnight. After fixation, the lungs were thoroughly washed with water for several hours. Subsequently, the lungs underwent a dehydration process involving sequential treatments with increasing concentrations of ethanol: 70% ethanol (overnight), 80% ethanol (2 h), 85% ethanol (2 h), 90% ethanol (45 min), 95% ethanol (20 min), and 100% ethanol (10 min, twice). Following ethanol treatment, the samples were immersed in xylene for 4 min, repeated twice. Finally, the specimens were embedded in paraffin, sectioned into 5 µm-thick slices, and stained using standard Hematoxylin & Eosin (H&E).

### Macrophages in vitro treatment

Peritoneal macrophages or bone marrow-derived macrophages were seeded in 6-well plates at a density of 1.2–2 × 10^6^ cells/well or in 96-well ultra-low attachment-bottom plates at a density of 2–4 × 10^5^ cells/well. Macrophages were allowed to adhere to the wells and rest for 6 h. Subsequently, the macrophages were pre-treated with either DMSO, CH223191 (MCE, HY-12684), STAT5-IN-1 (MCE, HY-101853-5mg), or IQDMA (Abcam, ab141192) for 2 h. After pre-treatment, the macrophages were cultured in RPMI 1640 complete medium supplemented with the respective compounds (DMSO, CH223191, STAT5-IN-1, or IQDMA) and tumor-conditioned medium with the same compounds for continued culture. For MG132 (Sigma, C2211-5MG) or CHX (Sigma-Aldrich, C7698-1G) treatment, the respective compound was added for 4 h before cells were collected for analysis.

### Flow cytometry and cell sorting

Single-cell suspensions were prepared. All antibodies were diluted at a 1:100 ratio. Samples were incubated at 4 °C, protected from light, for 30 min with the indicated fluorescent antibodies for surface molecular staining. After staining, samples were washed with PBS to remove excess antibodies. Prior to staining macrophages, incubate the cells with anti-mouse CD16/CD32 antibody (eBioscience, 14-0161-86) for 15 min at room temperature to prevent Fc receptor binding in the subsequent staining steps. For staining of the intranuclear protein Foxp3, cells were fixed and permeabilized overnight at 4 °C using the Intracellular Fixation & Permeabilization Buffer Set (Invitrogen, 88-8824-00), following staining of the proteins on the cell membrane surfaces. Subsequently, staining with the Foxp3 antibody was performed at 4 °C for 30 min. Comprehensive information on the antibodies can be found in Supplementary Table 1. For cell sorting, a Moflo Astrios EQ cell sorter (Beckman Coulter) and Beckman CytoFLEX SRT were employed. Flow cytometry data were acquired on a CytoFLEX LX (Beckman Coulter) and analyzed using FlowJo software.

### Mouse serum preparation

Mice were anesthetized, and cardiac blood samples were collected using syringes. These samples were transferred into 1.5 mL Ep tubes and left at room temperature for more than 2 h. They were then centrifuged at 4000 rpm for 10 min at room temperature, and the top liquid layers were carefully extracted. This process was repeated once more, and the final top liquid layers were collected and stored at −80 °C.

### Preparation of mouse lung homogenate

Lung tissue (~0.1 g) was taken directly (without washing with PBS), combined with magnetic beads, and flash-frozen in liquid nitrogen. For each sample, 500 µl of pre-cooled PBS containing PMSF was added, and the tissue was ground for 1 min. Subsequently, the samples were centrifuged at 12,000 rpm at 4 °C for 15 min, and the supernatant was carefully removed.

### Preparation of 4T1-CM

4T1 cells were plated on 10 cm^2^ dishes and cultured in 10 ml RPMI 1640 complete medium. The supernatants were collected when cell confluency reached 90–95%. Supernatants were then centrifuged at 2000 rpm for 5 min and sterilized using 0.22 μm filters. To stimulate macrophages, 4T1-CM was mixed with RPMI 1640 complete medium at a 3:7 ratio.

### Treg cell differentiation in vitro

For in vitro Treg cell differentiation, splenic CD4^+^ T cells were maintained for 3 days in RPMI 1640 supplemented medium, in the presence of anti-CD3 (5 µg/mL) and anti-CD28 (2 µg/mL) antibodies, IL-2 (50 ng/mL), and TGF-β (5 ng/mL). To test the influence of macrophages on Treg cell differentiation, macrophages were plated in 96-well flat-bottom plates at different densities (0.3125 × 10^5^, 0.625 × 10^5^, or 1.25 × 10^5^ cells/well) and maintained in complete RPMI 1640 medium supplemented with LPS, IL-4, or 4T1-CM (30% v/v). Forty-eight hours later, macrophages were washed with PBS twice and co-cultured with CD4^+^ T cells (5 × 10^5^ or 1 × 10^6^/well) under Treg cell differentiation medium for 3 days, with anti-PD-L1 antibody or Isotype (Bio X cell, BE0101) added.

### Immunofluorescence staining

Cells on slides were treated with 4% paraformaldehyde for 10 min at room temperature. Fixed cells were then incubated with PBS containing 0.1% Triton^TM^ X-100 for 10 min to increase cell permeability. Subsequently, cells were blocked with PBS containing 2% BSA for 10 min. The AHR monoclonal antibody (Invitrogen, MA1-514) was diluted in PBS containing 0.1% BSA at a 1:200 ratio. The AHR antibody was added to the cells and incubated overnight at 4 °C. The AHR antibody was then aspirated, and the cells were washed 3 times with PBS for 5 min each time. Samples were then incubated with a fluorescent secondary antibody diluted at a 1:2000 ratio in PBS containing 0.1% BSA at room temperature for 45 min. After washing with PBS 3 times, slides were sealed using Antifade mountant with DAPI (Invitrogen, P36931).

For immunofluorescence staining of mouse lung tissue, samples were fixed in 4% paraformaldehyde overnight, sequentially dehydrated with different concentration of sucrose, and embedded in OCT (Sakura Tissue-Tek). Nonspecific binding of antibodies was blocked with 5% BSA for 30 min. The F4/80 monoclonal antibody (CST, 70076T) and PD-L1 monoclonal antibody (Invitrogen, 16-5983-82) were diluted in PBS containing 1% BSA at a 1:200 ratio. Sections were incubated with diluted antibodies overnight at 4 °C. The antibodies were then aspirated, and the sections were washed 3 times with PBS. Sections were then incubated with a fluorescent secondary antibody diluted at a 1:2000 ratio in PBS containing 1% BSA at room temperature for 45 min. After washing with PBS 3 times, slides were sealed using Antifade mountant with DAPI (Invitrogen, P36931).

For immunofluorescence staining of human lung tissue, paraffin sections were subjected to antigen retrieval in a citrate acid buffer at 95 °C for 20 min. Sections were incubated with 3% hydrogen peroxide for 10 min, and washed 3 times with PBS, then blocked with 5% BSA for 30 min. F4/80 monoclonal antibody (CST, 70076T) and AHR antibodies (NOVUS, NB100-128SS) were diluted in PBS containing 1% BSA at a 1:400 ratio. Sections were incubated with diluted antibodies at room temperature for 1 h and then washed 3 times with PBS. The HRP-labeled antibody was incubated at room temperature for 30 min. After washing with PBS three times, the slides were sealed using Antifade mountant with DAPI (Invitrogen, P36931).

### Immunohistochemistry

For immunostaining, paraffin sections were subjected to antigen retrieval in a citrate acid buffer at 95 °C for 20 min. Sections were incubated with 3% hydrogen peroxide for 10 min, then washed 3 times with PBS and blocked with 5% BSA for 30 min. The sections were incubated with antibodies against Foxp3 (Thermo Fisher, 14-5773-82; 1:200) to detect Treg cells. Following this, sections were treated with suitable secondary antibodies. Mayer’s hematoxylin was used to counterstain the slides.

### Bulk RNA-seq

Alveolar macrophages (AM) were purified, and total RNA was extracted using the Trizol reagent (Invitrogen). Sequencing libraries were generated using the NEBNext Ultra RNA library prep kit from Illumina (NEB), following the manufacturer’s instructions, and index codes were added to each sample to attribute sequences. Samples were then sequenced on an Illumina HiSeq platform. Hisat2 v2.0.5 was used to build the index of the reference genome and to align paired-end clean reads with the reference genome. FeatureCounts v1.5.0-p3 was used to count the number of reads mapped to each gene. The fragments per kilobase of transcript per million mapped reads (FPKM) of each gene were calculated based on the length of the gene and reads count mapped to this gene. Metadata are available at https://www.ncbi.nlm.nih.gov/geo/query/acc.cgi?acc=GSE277589.

### ChIP-qPCR

A total of 1 × 10^7^ cells were prepared for each sample. Samples were then subjected to the subsequent steps according to the instructions provided in the ChIP-IT® PBMC kit (Active Motif, 53040). After obtaining ChIP-DNA, real-time fluorescence quantitative PCR was performed to assess AHR binding. The AHR binding region to the *Pdl1* promoter was predicted using the JASPAR database. The sequences of primers utilized for ChIP-qPCR can be found in Supplementary Table 2.

### CRISPR-KO cell line construction

For the stable transfection of 4T1 cells, the cells were electroporated with the lentiCRISPR v2 (Addgene, 52961) plasmids. In brief, 1 × 10^6^ 4T1 cells were suspended in 100 µl of electroporation buffer with 2 µg of the lentiCRISPR v2 plasmids. Subsequently, the 4T1 cells were electroporated and cultured in RPMI 1640 complete medium containing 3 μg/ml puromycin (MCE, HY-B1743A-10mg) for selection. After culturing with puromycin for 4 days, the knockout efficiency was confirmed by flow cytometry analysis. To establish stable 4T1 cells carrying GM-CSF deletion, 3 μg of Cas9-EGFP proteins (GenScript, Z03393-100) and 25 pmol of synthesized small guide RNAs (sgRNAs) were directly electroporated into 4T1 cells. After incubation for 48 h, a single EGFP-positive cell was selected using flow cytometry. All gRNA sequences used for CRISPR-KO experiments are listed in Supplementary Table 3. lentiCRISPR v2 (Addgene, 52961) plasmid was generously provided by Dr. Yuexiang Wang from the Shanghai Institute of Nutrition and Health, University of Chinese Academy of Sciences.

### ELISA assay

The concentration of mouse GM-CSF in the homogenate supernatant from the lungs or serum of 4T1 tumor-bearing mice was determined using the ELISA kit (eBioscience, BMS612) following the manufacturer’s protocol.

### Overall survival of breast cancer patients with *AHR* expression

Overall survival data of breast cancer patients with *AHR* expression were obtained from 2976 patients curated by Kaplan-Meier Plotter based on RNA-seq. For breast cancer patients, inclusion criteria did not impose limitations regarding lymph node involvement, ER expression, PR expression, HER2 expression, KI67 index, Nottingham histologic grading, or classification by PAM50 subtype. Overall survival data for triple-negative breast cancer (TNBC) patients were obtained from 126 TNBC patients. ER-negative, PR-negative, and HER2-negative statuses were used to restrict the analysis to TNBC patients. Patients were split using the auto-selected best cutoff.

### Gene expression and infiltrating immune cell correlation analysis

Gene expression and infiltrating immune cell correlation analysis were performed using the TIMER2.0 online tool.^59^ The correlation of *AHR* expression with *CD274* and the correlation of *AHR* expression or *CD274* with Treg infiltration in breast cancer (n = 1100) and basal-like breast cancer (n = 191) were analyzed. The correlation of *AHR* expression or *CD274* with Treg infiltration was assessed using the quanTIseq method.

### Statistical analysis

Data were presented as mean ± SEM or symbols & lines, as specified in respective figure legends, and were analyzed using Prism 9. Statistical significance was assessed using various tests, including paired two-tailed t-test, unpaired two-tailed t-test, log-rank (Mantel-Cox) test, Pearson correlation analysis, ordinary one-way ANOVA or Mann Whitney test, as appropriate. Significant differences between or among groups are indicated as follows: *p < 0.05; **p < 0.01; ***p < 0.001; ****p < 0.0001; ns, no significance.

## Supplementary information

Supplementary Materials

## Data Availability

The accession number for mRNA-Seq datasets reported in this paper is GEO: GSE277589. The authors verify that all other data supporting the findings of this study are provided in the article and its Supplemental Information files.
