# Supplementary material for: Macrophages promote pre-metastatic niche formation of breast cancer through aryl hydrocarbon receptor activity
Source: Signal Transduct Target Ther. 2024 Dec 18;9:352. doi: 10.1038/s41392-024-02042-5 (PMC11652640; doi:10.1038/s41392-024-02042-5)
Supplement: Supplementary file 1 — Supplementary Materials [file 41392_2024_2042_MOESM1_ESM.doc]

Supplementary Materials for

Macrophages promote pre-metastatic niche formation of breast cancer through aryl hydrocarbon receptor activity

Xu Jiang^1,5^, Jiaqi Wang^1,5^, Liangyu Lin^1^, Liming Du^1^, Yayun Ding^3^, Fanjun Zheng^1^, Hongzhen Xie^1^, Yu Wang^1^, Mingyuan Hu^1^, Benming Liu^1^, Muhan Xu^1^, Jingjie Zhai^1^, Xuefeng Wang^1^, Jiayin Ye^1^, Wei Cao^1^, Chao Feng^2^, Jingyi Feng^1^, Zongliu Hou^4^, Mingyao Meng^4^, Ju Qiu^1^, Qing Li^1,*^, Yufang Shi^1, 2,*^, Ying Wang^1,*^

*Correspondence to: qingli@sibs.ac.cn (Q.L.); yfshi@suda.edu.cn (Y.S.); yingwang@sibs.ac.cn (Y.W.).

**This PDF file includes:**

Supplementary Figures 1 to 7

Supplementary Tables 1 to 4

**Other Supplementary Materials for this manuscript include the following:**

Uncropped western blots

**Supplementary Figures**


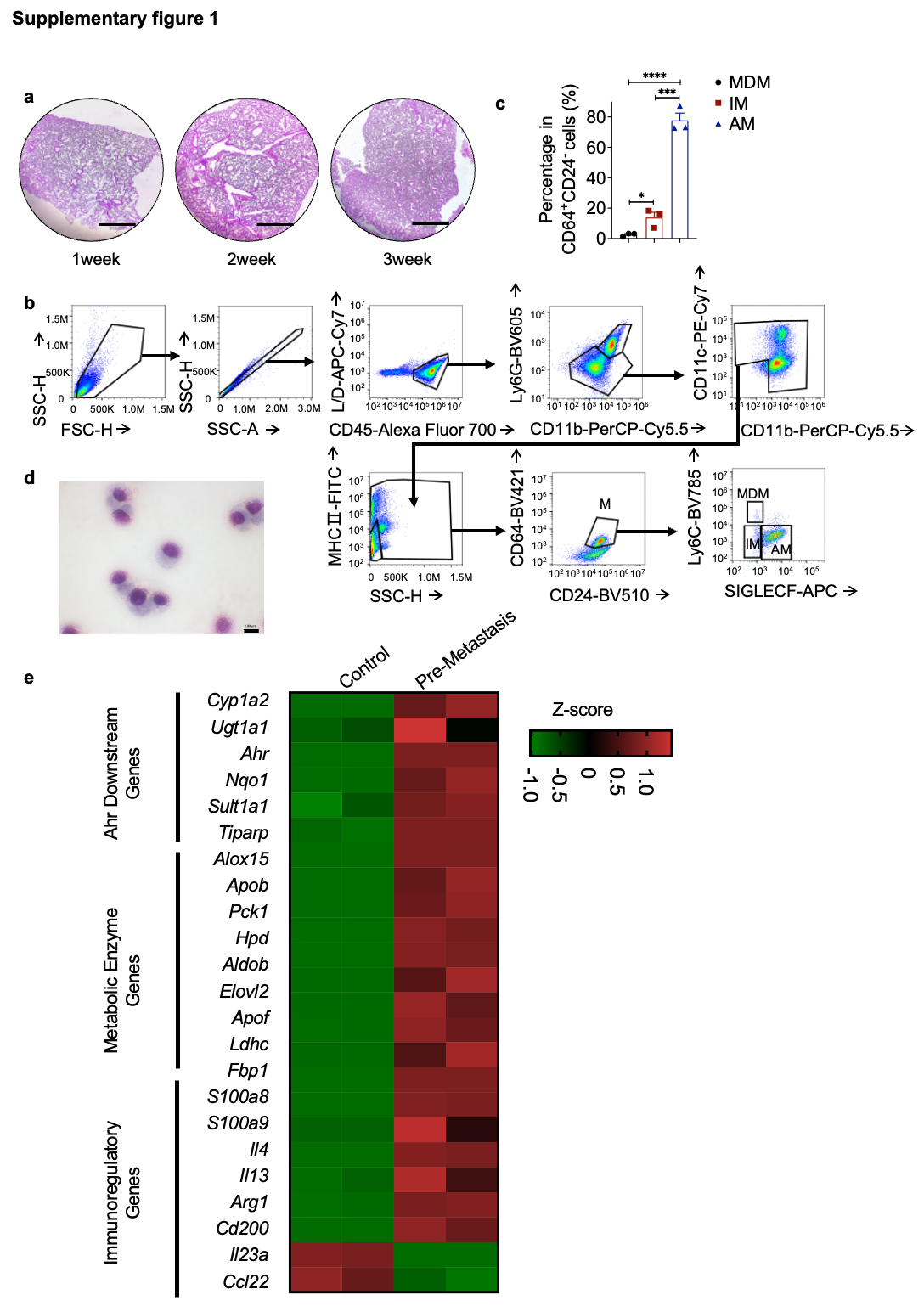


**Supplementary figure 1.** **AMs are the dominant macrophage subpopulation in the lung PMN of breast cancer. a** H&E staining of lung sections from tumour-bearing mice at the indicated time. Scale bars, 910 µm. **b** Gating strategy for macrophage identification in the lungs. **c** Flow cytometry analysis of the frequency of AMs, IMs, and MDMs in the lung macrophages of WT mice with 4T1 cells inoculation for 2 weeks (n=3). **d** Giemsa staining of CD45^+^ CD11b^-^ CD11c^+^ SiglecF^+^ macrophages isolated from the lungs of mice inoculated with 4T1 cells for 14 days. Scale bar, 100 µm. **e** Heatmap of the genes related to *Ahr* and its downstream targets, metabolic enzymes, and immunoregulation in AMs from control mice and mice inoculated with 4T1 tumour for 14 days (n=2 for each group). Data are analyzed by unpaired two-tailed t-test (**c**) and presented as mean ± SEM. *p<0.05, ***p<0.001, ****p<0.0001.


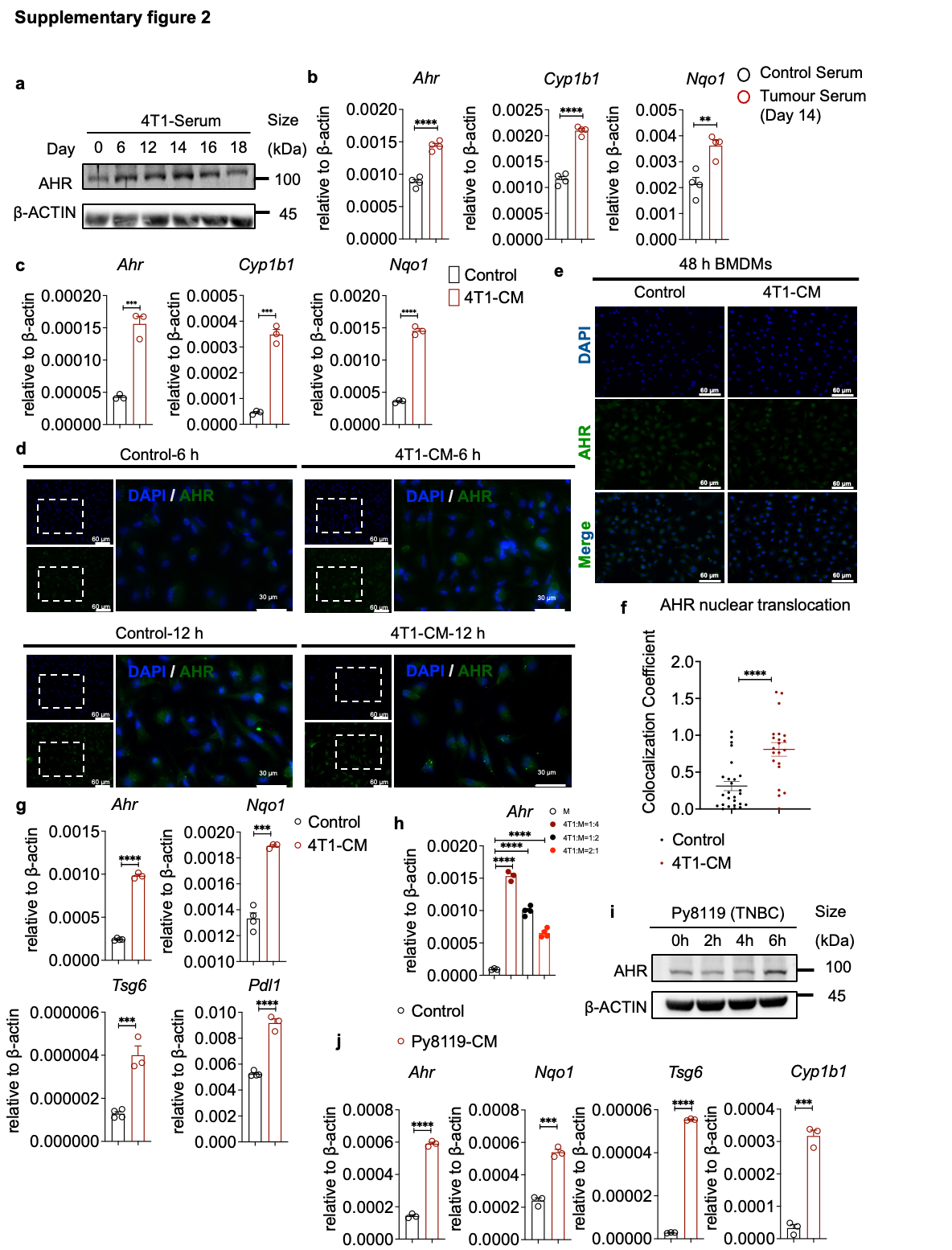


**Supplementary figure 2. AHR activity in macrophages is influenced by breast cancer. a** AHR levels in macrophages treated with serum from mice inoculated with 4T1 tumour for the indicated time**.** **b** mRNA levels of *Ahr* and its target genes *Cyp1b1* and *Nqo1* in BMDMs treated with serum from naïve mice and mice inoculated with 4T1 cells for 14 days (n=4 for each group, repeated 3 times). **c** mRNA levels of *Ahr*, *Cyp1b1*, and *Nqo1* in peritoneal macrophages treated with 4T1-conditioned medium (CM) for 48 h (n=3 for each group, repeated 3 times). **d** Representative immunofluorescent images showing AHR nuclear translocation in peritoneal macrophages treated with or without 4T1-CM for 6 h and 12 h. Scale bar in the merged picture is 30 µm. **e** Representative immunofluorescent images showing AHR nuclear translocation in BMDMs treated with or without 4T1-CM for 48 h. Scale bars, 60 µm. **f** Statistical analysis of AHR nuclear translocation in BMDMs treated with 4T1-CM or control medium (n≥21 for each group). **g** mRNA levels of *Ahr*, *Nqo1*, *Tsg6*, and *Pdl1* in AMs treated with 4T1 conditioned medium (n≥3 for each group). **h** mRNA expression of *Ahr* in peritoneal macrophages co-cultured with 4T1 cells at indicated ratios (n≥3 for each group, repeated 3 times). **i** Western blotting analysis of AHR levels in peritoneal macrophages treated with Py8119-conditioned medium (CM) for the indicated time. **j** mRNA levels of *Ahr*, *Nqo1*, *Tsg6,* and *Cyp1b1* in peritoneal macrophages treated with Py8119-CM for 48 h (n=3 for each group). Data are analyzed by unpaired two-tailed t-test (**b**, **c**, **f**, **g**, **h**, **j**) and presented as mean ± SEM. **p<0.01, ***p<0.001, ****p<0.0001.


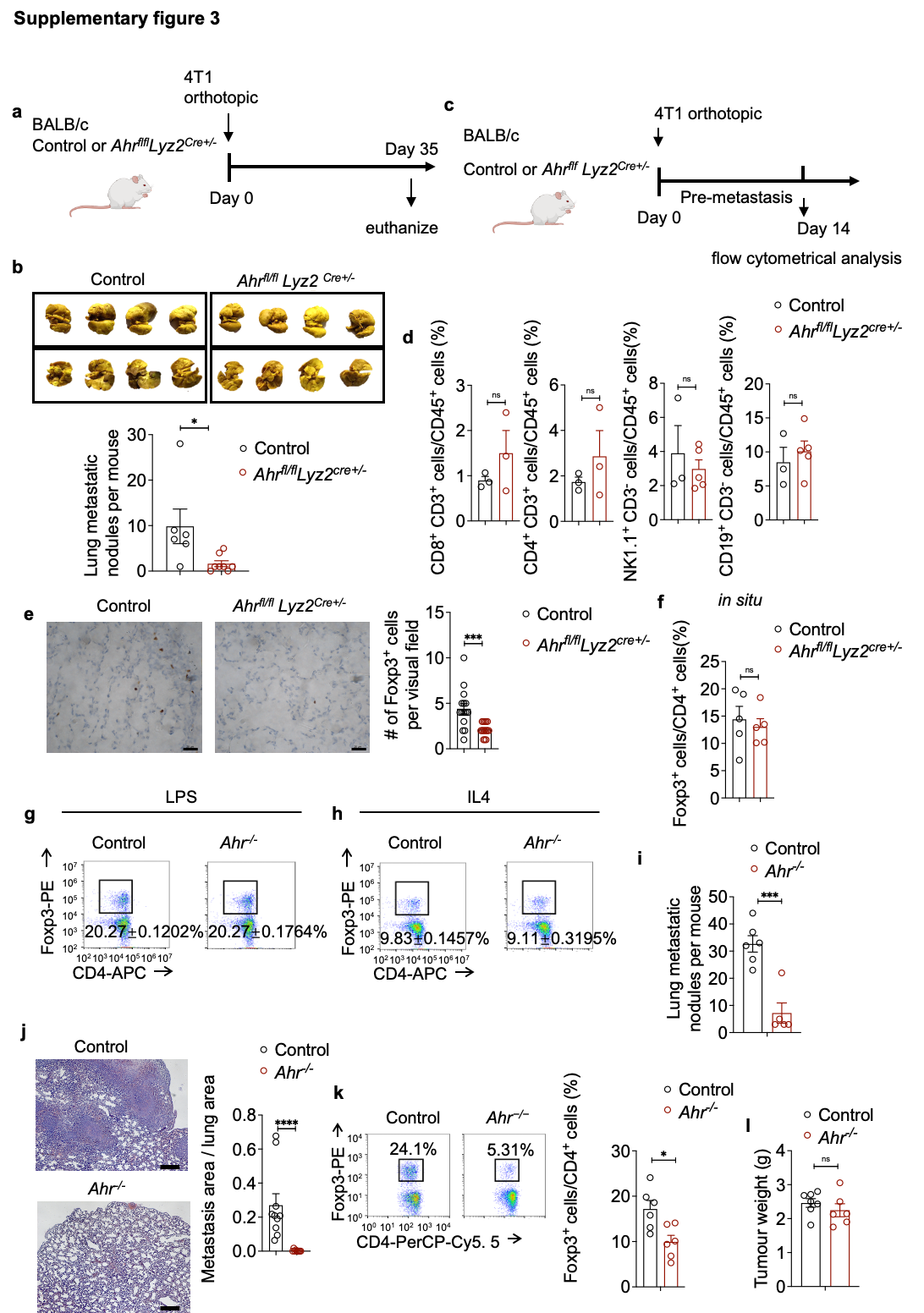


**Supplementary figure 3. The influence of AHR on breast cancer progression and immune cell profile.** **a** Schematic graph showing the establishment of 4T1 breast cancer lung metastasis (created with BioRender.com). **b** Wild-type (Control) and *Ahr^flfl^Lyz2^Cre+/-^*mice were inoculated with Py8119 cells in the mammary gland fat pad for 7 weeks. Picric acid-stained lung tissues and the number of macroscopic metastases in the lungs (n=6 for control group, n=8 for *Ahr^flfl^Lyz2^Cre+/-^*group) were assessed. **c** Schematic representation of the PMNs in the lungs of 4T1 breast cancer models in *Ahr^flfl^Lyz2^Cre-/-^* (Control) and *Ahr^flfl^Lyz2^Cre+/-^* mice (created with BioRender.com). **d** Flow cytometry analysis on the frequency of CD8^+^ T cells, CD4^+^ T cells, NK cells and B cells in the lungs of *Ahr^flfl^Lyz2^Cre-/-^* (Control) and *Ahr^flfl^Lyz2^Cre+/-^* bearing 4T1 tumour for 14 days (n≥3 for each group). **e** Foxp3 staining and statistical analysis of the lungs of *Ahr^flfl^Lyz2^Cre-/-^*(Control) and *Ahr^flfl^Lyz2^Cre+/-^* mice inoculated with 4T1 cells for 14 days (n=15 for each group). Scale bar, 20 μm. **f** Flow cytometry analysis of Treg frequency in primary tumours of *Ahr^flfl^Lyz2^Cre-/-^* (Control) and *Ahr^flfl^Lyz2^Cre+/-^* mice (n=5 for each group, repeated 3 times). **g**, **h** Representative flow cytometry plots depicting Treg cell differentiation. BMDMs from wild-type (Control) or *Ahr^-/-^* mice were pretreated with LPS (**g**) (n=3 for each group, repeated twice) or IL4 (**h**) (n=3 for each group) for 2 days and co-cultured with CD4^+^ T cells under Treg differentiation conditions for 3 days. **i**-**l** Lung metastatic nodules(n≥5 for each group) (**i**), H&E staining of lung sections and metastatic tumour burden quantification (n≥9 for each group) (**j**), Foxp3^+^ Treg cell percentages in the lungs (**k**) and primary tumour weight (**l**) of wild-type (control) and *Ahr*^-/-^ mice bearing 4T1 tumour (n=5-7 for each group, repeated 2-4 times). Scale bars, 400 µm. Data are analyzed by unpaired two-tailed t-test (**b**, **d**, **f**, **i**, **k**, **l**) or descriptive statistics (**g**, **h**) or Mann Whitney test (**e**, **j**) and presented as mean ± SEM. *p<0.05, ***p<0.001, ****p<0.0001, ns, no significance.


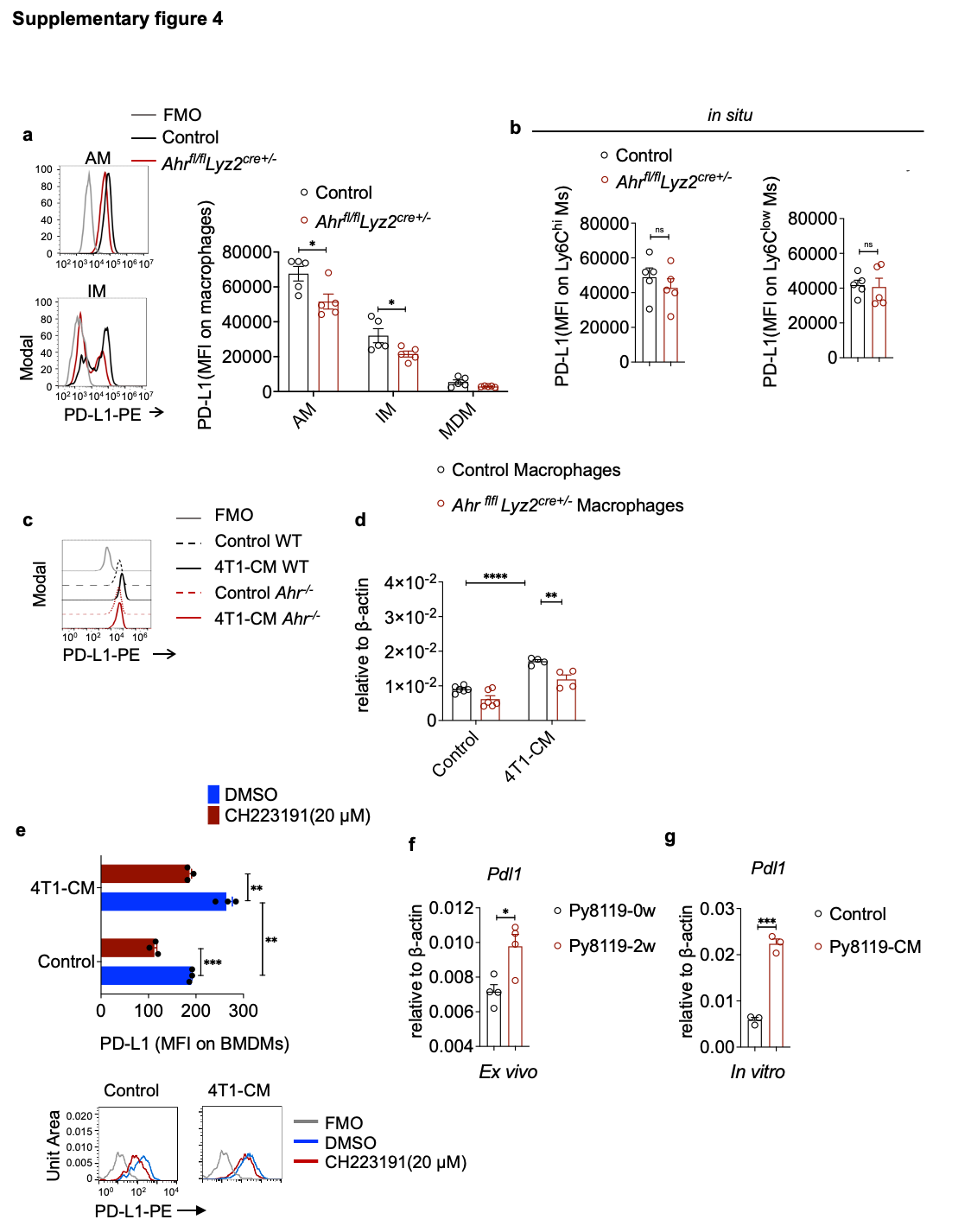


**Supplementary figure 4.** **AHR regulates PD-L1 expression on macrophages during breast cancer progression. a** PD-L1 expression on AMs, IMs, and MDMs from *Ahr*^flfl^*Lyz2*^Cre-/-^ (Control) and *Ahr*^flfl^*Lyz2*^Cre+/-^ mice (n=5). **b** PD-L1 expression on CD45^+^ CD11b^+^ F4/80^+^ MHCII^+^ Ly6C^hi^ cells and CD45^+^ CD11b^+^ F4/80^+^ MHCII^+^ Ly6C^low^ cells from primary tumours of *Ahr^flfl^Lyz2^Cre-/-^* (Control) and *Ahr^flfl^Lyz2^Cre+/-^* mice bearing 4T1 tumour was analyzed by flow cytometry (n=5 for each group, repeated 3 times). **c** Histogram showing PD-L1 expression on peritoneal macrophages from wild-type (WT) and *Ahr^-/-^*mice, with or without 4T1-CM treatment for 12 h. **d** mRNA levels of *Pdl1* in BMDMs (n≥4 for each group) from *Ahr^flfl^Lyz2^Cre-/-^* (Control) and *Ahr^flfl^Lyz2^Cre+/-^* mice, treated with 4T1-CM for 48 h. **e** PD-L1 expression on WT BMDMs treated with 4T1-CM for 48 h, in the presence or absence of CH223191 (20 μM) (n=3 for each group, repeated twice). **f** mRNA levels of *Pdl1* in F4/80^+^ macrophages from the lungs of Py8119-bearing mice (n=4 for each group). **g** mRNA levels of *Pdl1* in peritoneal macrophages treated with Py8119-CM for 48 h (n=3 for each group). Data are analyzed by unpaired two-tailed t-test (**a**, **b**, **d**, **e**, **f**, **g**) and presented as mean ± SEM. *p<0.05, **p<0.01, ***p<0.001, ****p<0.0001, ns, no significance.


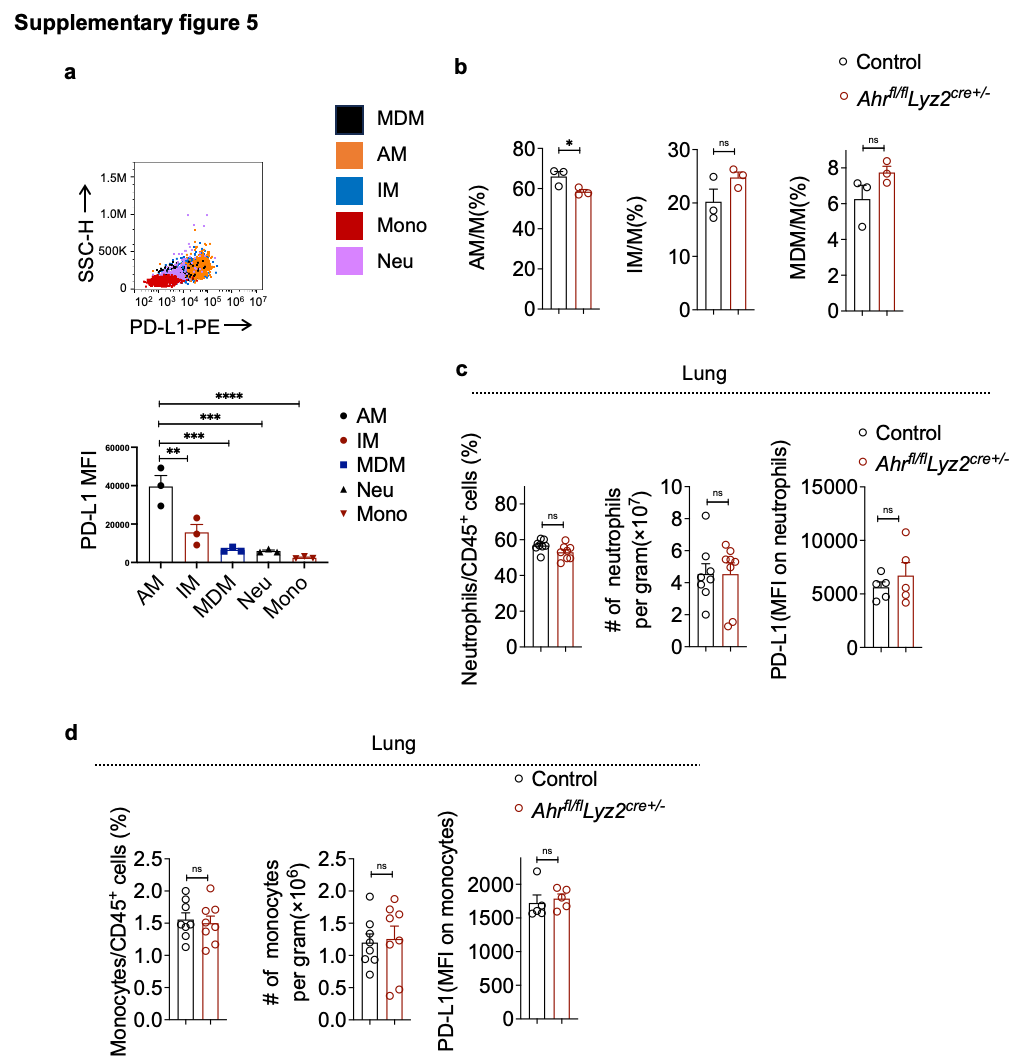


**Supplementary figure 5. AMs are the major population of lung macrophages expressing PD-L1 in the PMN. a** PD-L1 expression on AMs, IMs, MDMs, neutrophils, and monocytes in the lungs of WT mice inoculated with 4T1 cells for 2 weeks (n=3 for each group). **b** Flow cytometry analysis on AMs, IMs, and MDMs in the lungs of *Ahr^flfl^Lyz2^Cre-/-^*(Control) and *Ahr^flfl^Lyz2^Cre+/-^*mice inoculated with 4T1 cells in the mammary gland fat pad for 2 weeks. (n=3 for each group). **c**, **d** Flow cytometry analysis of the frequency, cell number, and PD-L1 expression of neutrophils (**c**) and monocytes (**d**) in the lungs of *Ahr^flfl^Lyz2^Cre-/-^* (Control) and *Ahr^flfl^Lyz2^Cre+/-^* mice inoculated with 4T1 cells for 14 days (n≥5 for each group). Data are analyzed by unpaired two-tailed t-test (**b**, **c**, **d**) or ordinary one-way ANOVA (**a**) and presented as mean ± SEM. *p<0.05, ns, no significance.


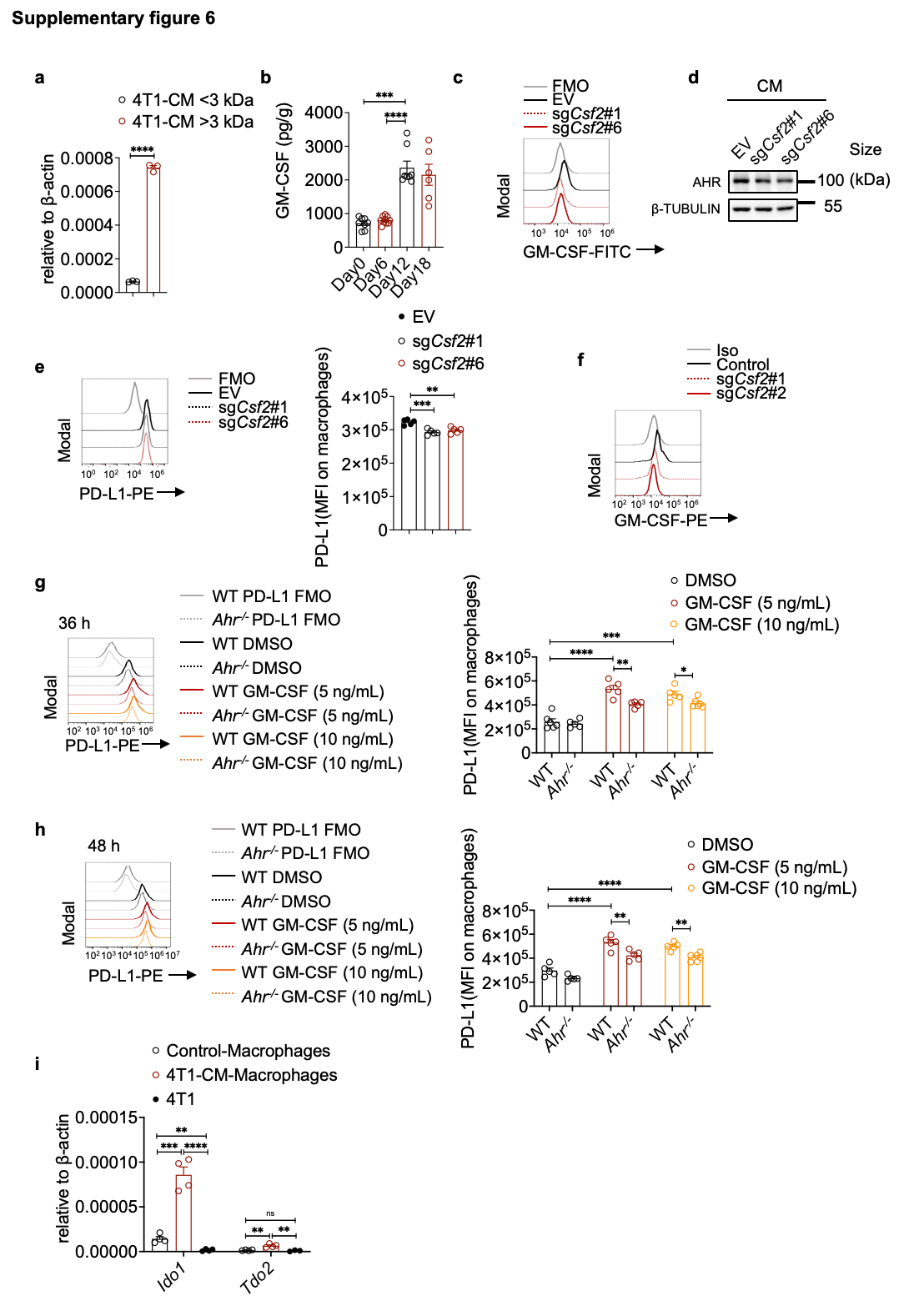


**Supplementary figure 6.** **AHR in macrophages is regulated by GM-CSF released from 4T1 cells. a** mRNA expression of *Ahr* in peritoneal macrophages treated with < 3-kDa or >3-kDa fraction of 4T1-CM (n=3 for each group). **b** Quantification of GM-CSF levels in lung tissue of mice bearing 4T1 tumour using ELISA (n≥6 for each group). **c** Flow cytometry analysis on GM-CSF expression on empty sgRNA vector (EV) and sgCsf2 transfected 4T1 cells (4T1 cells were transfected with lentiCRISPR v2 plasmids). **d** AHR expression in peritoneal macrophages treated with CM (30% v/v) from EV control or sg*Csf2* 4T1 cells (repeated twice). **e** Flow cytometry analysis of PD-L1 expression on peritoneal macrophages treated with EV 4T1-CM or sg*Csf2* 4T1-CM for 36 h. The bar graph shows MFI of PD-L1 for each group of macrophages (n=5 for each group). **f** Histogram showing GM-CSF expression on control (4T1 cells transfected with Cas9 protein) and sg*Csf2* 4T1 cells (4T1 cells transfected with single-guide RNA and Cas9 protein). **g** Histogram showing PD-L1 expression on WT or *Ahr^-/-^*peritoneal macrophages treated with or without GM-CSF (5 or 10 ng/mL) for 36 h (n≥5 for each group). The bar graph shows MFI of PD-L1 on each group of macrophages. **h** Flow cytometry analysis of PD-L1 expression on WT and *Ahr^-/-^* peritoneal macrophages treated with GM-CSF (5 or 10 ng/mL) for 48 h. The bar graph shows MFI of PD-L1 for each group of macrophages (n≥5 for each group). **i** mRNA expression of *Ido1* and *Tdo2* in 4T1 cells and peritoneal macrophages treated with or without 4T1-CM (n≥3 for each group). Data are analyzed by unpaired two-tailed t-test (**a**, **e**, **g**, **h, i**) or Mann-Whitney test (**b**) and presented as mean ± SEM. *p<0.05, **p<0.01, ***p<0.001, ****p<0.0001, ns, no significance.


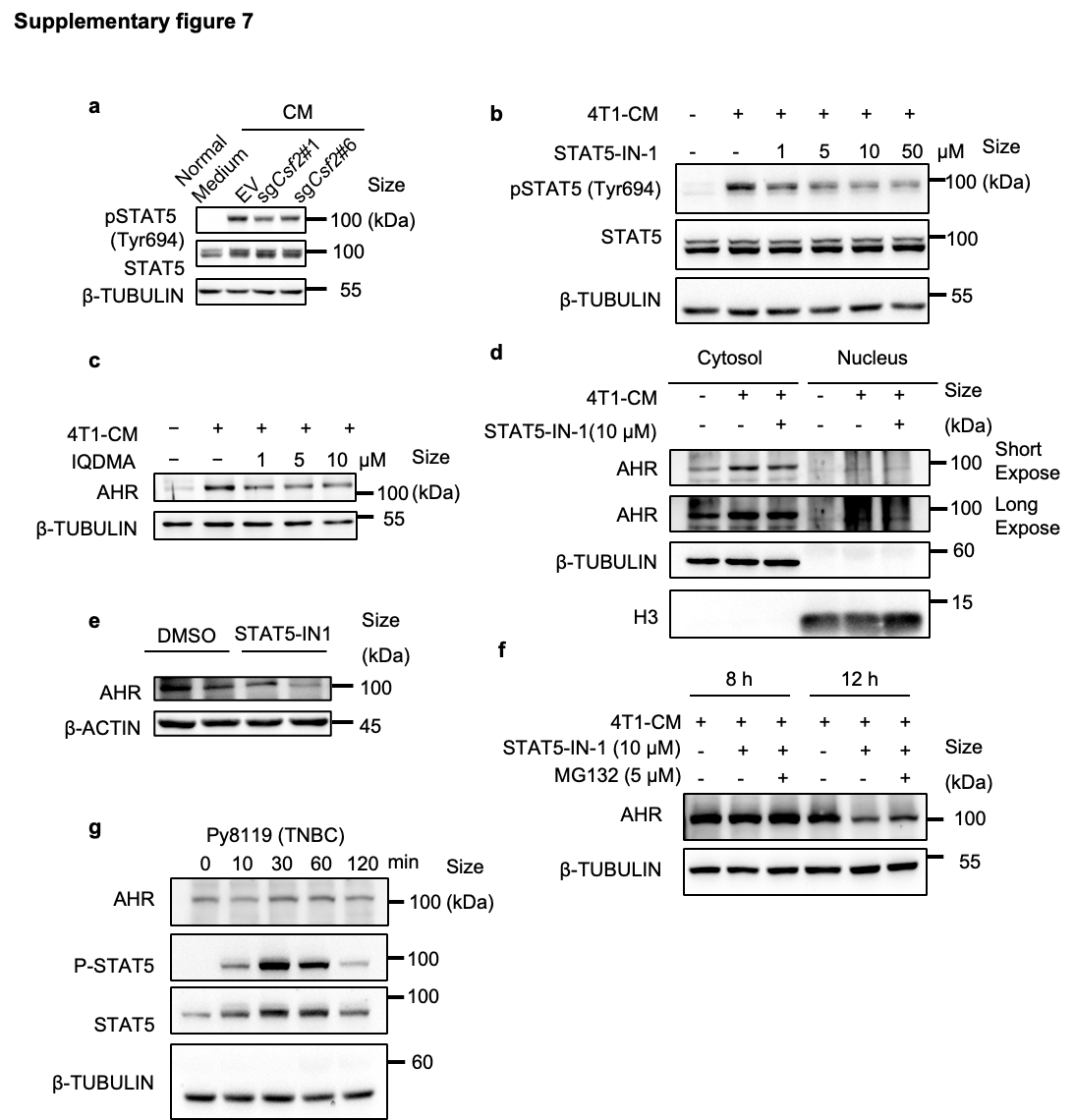


**Supplementary figure 7. GM-CSF-induced STAT5 signaling enhances AHR stability** **a** Western blotting analysis of pSTAT5 and STAT5 expressions in peritoneal macrophages cultured with conditioned medium (CM) from empty vector (EV)-transfected 4T1 cells or *Csf2* knockout 4T1 cells for 60 min (repeated twice). **b** Efficiency of STAT5-IN-1 in suppressing STAT5 activation in macrophages. Phosphorylated STAT5 expression was detected in peritoneal macrophages exposed to different concentrations of STAT5-IN-1. **c** Western blotting analysis of AHR expression in IQDMA-treated peritoneal macrophages, with or without 4T1-CM treatment for 24 h (repeated 3 times). **d** Western blot analysis of AHR expression in the cytoplasm and nucleus of macrophages treated by 4T1-CM, with or without STAT5 inhibitor. **e** Western blotting analysis of AHR levels in F4/80^+^ cells isolated from lung tissue of 4T1 tumour bearing mice treated with STAT5-IN1 or DMSO. **f** Western blotting analysis of AHR expression in peritoneal macrophages treated with 4T1-CM, with or without the addition of MG132 (5 μM) and STAT5-IN-1 (10 μM). **g** Western blotting analysis of AHR, pSTAT5, and STAT5 expression in peritoneal macrophages treated with Py8119-CM.

**
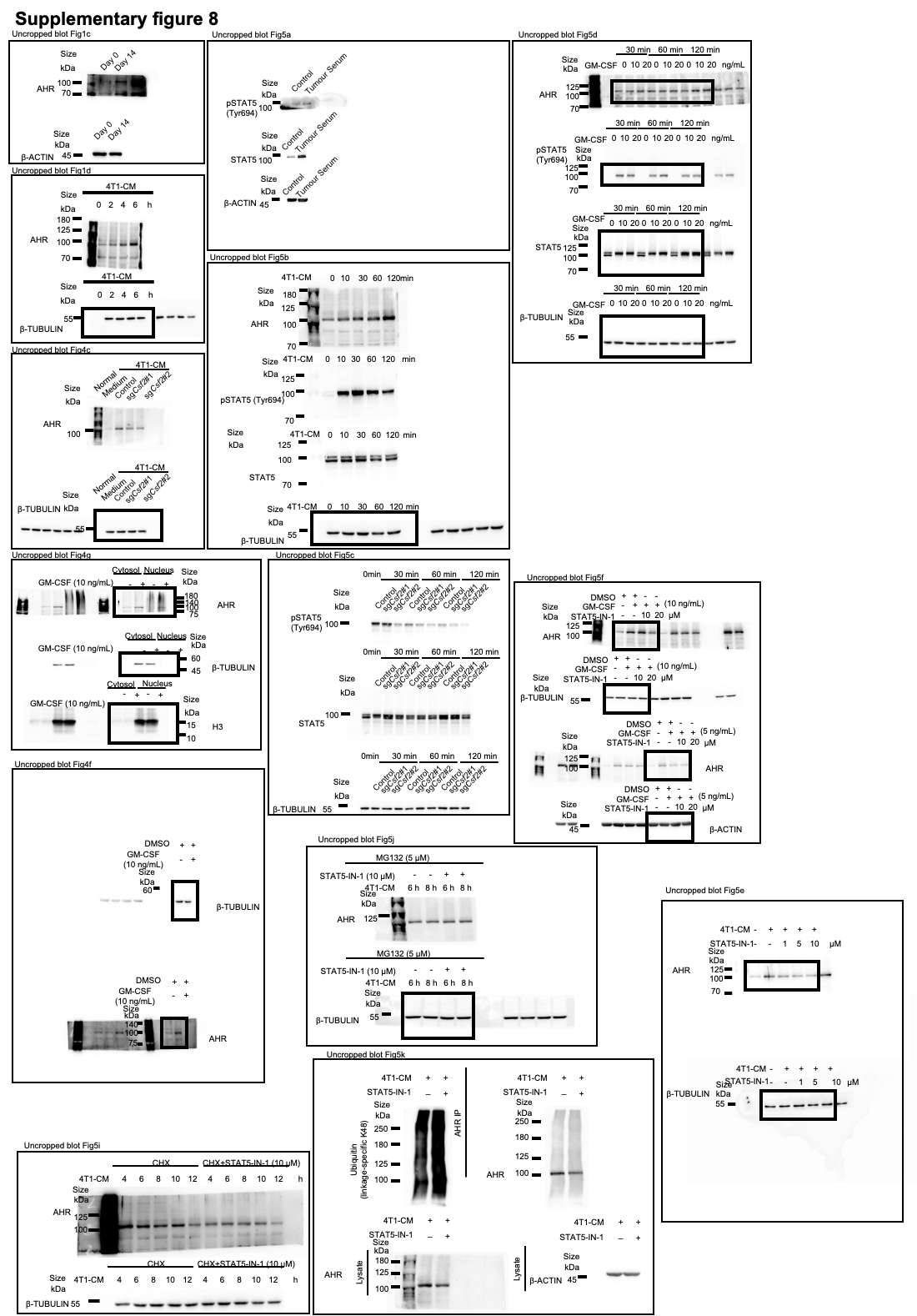
**

**Supplementary figure 8. Uncropped western blots for Figures.**

**
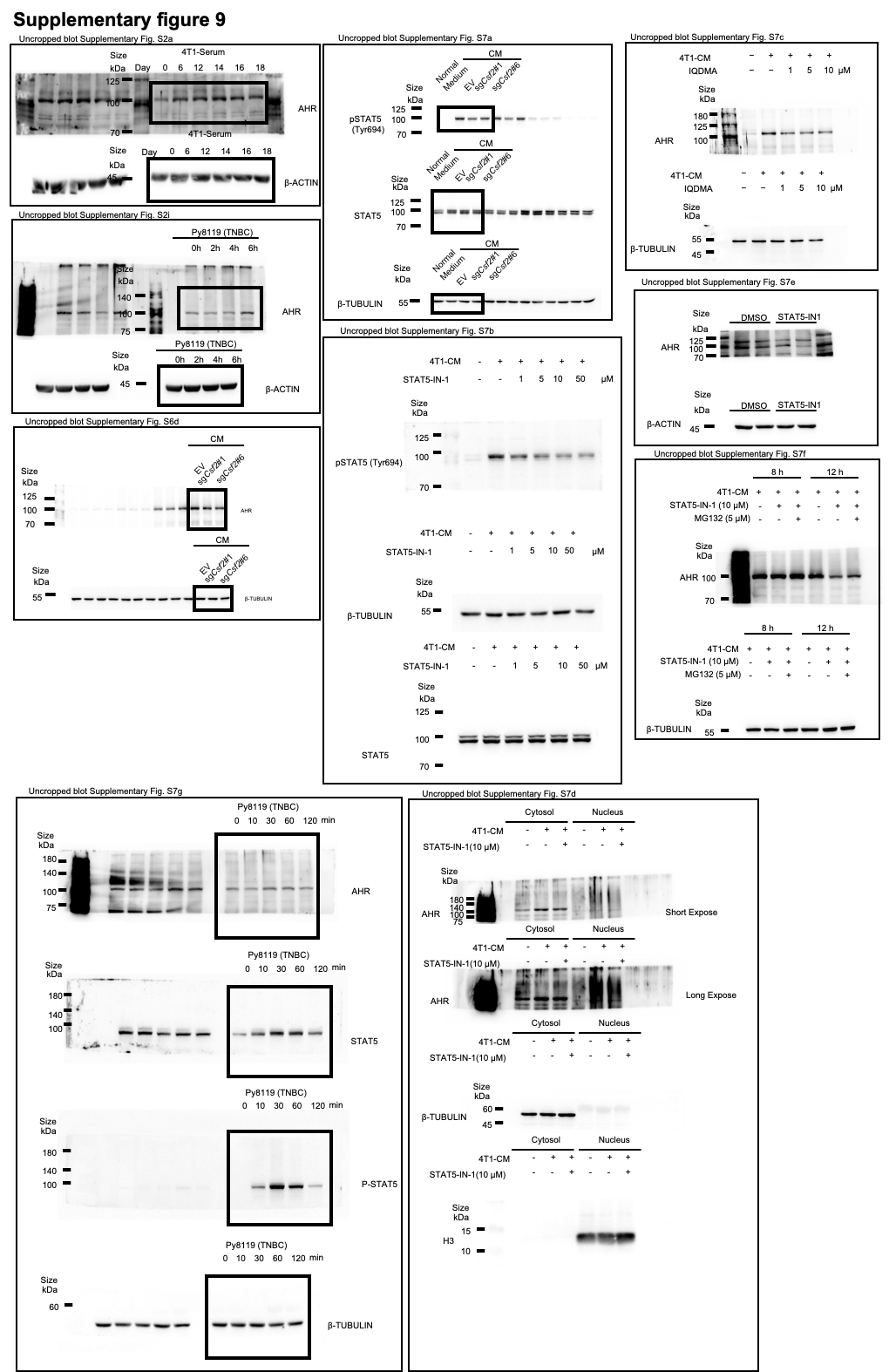
**

**Supplementary figure 9. Uncropped western blots for Supplementary figures.**

Supplementary Table 1. Antibody Information

| **Antibodies or reagents** | **Source** | **Identifier** |
| --- | --- | --- |
| Pacific Blue™ anti-mouse CD45 Antibody | BioLegend | 103126 |
| Rat IgG2a kappa Isotype Control (eBR2a), PE | eBioscience | 12-4321-81A |
| Rat IgG2a kappa Isotype Control (eBR2a), FITC | eBioscience | 11-4321-81 |
| GM-CSF Monoclonal Antibody (MP1-22E9), FITC | eBioscience | 11-7331-81 |
| GM-CSF Monoclonal Antibody (MP1-22E9), PE | eBioscience | 12-7331-82 |
| Brilliant Violet 785™ anti-mouse Ly-6C Antibody | BioLegend | 128041 |
| Brilliant Stain Buffer | BD | 563794 |
| Brilliant Violet 605™ anti-mouse Ly-6G | BioLegend | 127639 |
| MHC Class II (I-A/I-E) Monoclonal Antibody (M5/114.15.2), FITC | eBioscience | 11-5321-82 |
| Brilliant Violet 510™ anti-mouse CD24 Antibody | BioLegend | 101831 |
| Brilliant Violet 785™ anti-mouse CD8a Antibody | BioLegend | 100750 |
| Brilliant Violet 421™ anti-mouse CD64 (FcγRI) Antibody | BioLegend | 139309 |
| Alexa Fluor® 700 anti-mouse CD45 Antibody | BioLegend | 103128 |
| PerCP/Cyanine5.5 anti-mouse Ly-6G Antibody | BioLegend | 127616 |
| CD4 Monoclonal Antibody (RM4-5), PerCP-Cyanine5.5 | eBioscience | 45-0042-82 |
| CD45 Monoclonal Antibody (30-F11), PE | eBioscience | 12-0451-82 |
| CD274 (PD-L1, B7-H1) Monoclonal Antibody (MIH5), PE | eBioscience | 12-5982-83 |
| PE/Cyanine7 anti-mouse CD11c Antibody | BioLegend | 117318 |
| CD11b Monoclonal Antibody (M1/70), APC | ebioscience | 17-0112-83 |
| CD45 Monoclonal Antibody (30-F11), FITC | eBioscience | 11-0451-85 |
| Ly-6C Monoclonal Antibody (HK1.4), APC | eBioscience | 17-5932-82 |
| CD8a Monoclonal Antibody (53-6.7), FITC | eBioscience | 11-0081-85 |
| FOXP3 Monoclonal Antibody (FJK-16s), PE | eBioscience | 12-5773-82 |
| CD11b Monoclonal Antibody (M1/70), PerCP-Cyanine5.5 | eBioscience | 45-0112-82 |
| CD45 Monoclonal Antibody (30-F11), APC | eBioscience | 17-0451-83 |
| LIVE/DEAD Fixable Near-IR Dead Cell Stain Kit | eBioscience | L34976 |
| F4/80 Monoclonal Antibody (BM8), PerCP-Cyanine5.5 | eBioscience | 45-4801-82 |
| CD11b Monoclonal Antibody (M1/70), PE | eBioscience | 12-0112-83 |
| CD11b Monoclonal Antibody (M1/70), FITC | eBioscience | 11-0112-82 |
| CD4 Monoclonal Antibody (GK1.5), APC | eBioscience | 17-0041-83 |
| Brilliant Violet 510™ anti-mouse CD45.2 Antibody | BioLegend | 109838 |
| Alexa Fluor® 700 anti-mouse Ly-6G Antibody | BioLegend | 127622 |
| BUV496 Hamster Anti-Mouse CD11c | BD | 750483 |
| BV650 Rat Anti-Mouse CD24 | BD | 563545 |
| FITC anti-mouse CD4 Antibody | BioLegend | 100406 |
| F4/80 Monoclonal Antibody (BM8), FITC | eBioscience | 11-4801-85 |
| Brilliant Violet 510™ anti-mouse F4/80 Antibody | BioLegend | 123135 |
| Alexa Fluor® 647 anti-mouse CD3 Antibody | BioLegend | 100209 |
| Brilliant Violet 605™ anti-mouse CD4 Antibody | BioLegend | 100548 |
| PE anti-mouse CD3 Antibody | BioLegend | 100206 |
| FITC anti-mouse CD3ε Antibody | BioLegend | 100306 |
| F4/80 Monoclonal Antibody (BM8), PerCP-Cyanine5.5 | eBioscience | 45-4801-82 |
| Brilliant Violet 421™ anti-mouse CD45 Antibody | BioLegend | 103134 |
| AHR Antibody | NOVUS | NB100-128S |
| F4/80 (D2S9R) XP ® Rabbit mAb | Cell Signaling(CST) | 70076T |
| FOXP3 Monoclonal Antibody (FJK-16s), eBioscience | ThermoFisher | 14-5773-80 |

**Supplementary Table 2. The Primer Sequences Used for ChIP–qPCR**

| ***Pdl1* promoter** | **Forward primer** | **Reverse primer** | **Site on *Pdl1* locus** |
| --- | --- | --- | --- |
| *Pdl1* promoter | GCAGGGACAGTACCTTGCTT | GCAGGTTGGAATTTGCGGTT | -1166 |
| *Pdl1* promoter | CCCTGGAATTAAGCAGCCCT | CAAACGGCTAGAGACCCCTC | -2817 |
| *Pdl1* promoter | AGTACCTTGCTTCGGCAGAG | ATGCAGGTTGGAATTTGCGG | -1158 |

**Supplementary Table 3. All gRNA Sequences Used for CRISPR-KO Experiments**

| **sgRNA for plasmid** | **forward sequences 5'-3'** |
| --- | --- |
| mouse *csf2*#1 | CACCGGCTGTCTATGAAATCCGCAT |
| mouse *csf2*#2 | CACCGGGCTGTAGACCACAATGCCC |
| mouse *csf2*#6 | CACCGGGATGACATGCCTGTCACGT |
| **synthesized sgRNA** | **forward sequences 5'-3'** |
| mouse *csf2*#1 | **mG*mC*mU*rGrUrCrUrArUrGrArArArUrCrCrGrCrA rUrGrUrUrUrUrArGrArGrCrUrArGrArArArUrArGrCrA rArGrUrUrArArArArUrArArGrGrCrUrArGrUrCrCrGrU rUrArUrCrArArCrUrUrGrArArArArArGrUrGrGrCrArC rCrGrArGrUrCrGrGrUrGrCrU*mU*mU*mU** |
| mouse *csf2*#2 | **mG*mG*mC*rUrGrUrArGrArCrCrArCrArArUrGrCrC rCrGrUrUrUrUrArGrArGrCrUrArGrArArArUrArGrCrA rArGrUrUrArArArArUrArArGrGrCrUrArGrUrCrCrGrU rUrArUrCrArArCrUrUrGrArArArArArGrUrGrGrCrArC rCrGrArGrUrCrGrGrUrGrCrU*mU*mU*mU** |

**Supplementary Table 4. Information of clinical tissue samples**

| Sample Number | Sample character | Metastasis  (Yes or No) |
| --- | --- | --- |
| 1# | Lung tissue section/ Non-breast cancer | N |
| 2# | Lung tissue section/ Non-breast cancer | N |
| 3# | Lung tissue section/ Non-breast cancer | N |
| 4# | Lung tissue section/ Breast cancer | Y |
| 5# | Lung tissue section/ Breast cancer | Y |
| 6# | Lung tissue section/ Breast cancer | Y |
| 7# | Lung tissue section/ Breast cancer | Y |
| 8# | Lung tissue section/ Breast cancer | Y |
